# CDC42 deficiency leads to endometrial stromal cell senescence in recurrent implantation failure

**DOI:** 10.1093/humrep/deae246

**Published:** 2024-11-01

**Authors:** Xinyi Tang, Yingchun Zhu, Zhiwen Cao, Xiaoying Wang, Xinyu Cai, Yurun Tang, Jidong Zhou, Min Wu, Xin Zhen, Lijun Ding, Guijun Yan, Haibin Wang, Haixiang Sun, Ruiwei Jiang

**Affiliations:** Center for Reproductive Medicine and Obstetrics and Gynecology, Nanjing Drum Tower Hospital, Affiliated Hospital of Medical School, Nanjing University, Nanjing, China; Center for Molecular Reproductive Medicine, Nanjing University, Nanjing, China; Center for Reproductive Medicine and Obstetrics and Gynecology, Nanjing Drum Tower Hospital, Affiliated Hospital of Medical School, Nanjing University, Nanjing, China; Center for Molecular Reproductive Medicine, Nanjing University, Nanjing, China; Center for Reproductive Medicine and Obstetrics and Gynecology, Nanjing Drum Tower Hospital, Affiliated Hospital of Medical School, Nanjing University, Nanjing, China; Center for Molecular Reproductive Medicine, Nanjing University, Nanjing, China; Center for Reproductive Medicine and Obstetrics and Gynecology, Nanjing Drum Tower Hospital, Affiliated Hospital of Medical School, Nanjing University, Nanjing, China; Center for Molecular Reproductive Medicine, Nanjing University, Nanjing, China; Center for Reproductive Medicine and Obstetrics and Gynecology, Nanjing Drum Tower Hospital, Affiliated Hospital of Medical School, Nanjing University, Nanjing, China; Center for Molecular Reproductive Medicine, Nanjing University, Nanjing, China; Center for Reproductive Medicine and Obstetrics and Gynecology, Nanjing Drum Tower Hospital Clinical College of Nanjing Medical University, Nanjing, China; Center for Reproductive Medicine and Obstetrics and Gynecology, Nanjing Drum Tower Hospital, Affiliated Hospital of Medical School, Nanjing University, Nanjing, China; Center for Molecular Reproductive Medicine, Nanjing University, Nanjing, China; Center for Reproductive Medicine and Obstetrics and Gynecology, Nanjing Drum Tower Hospital, Affiliated Hospital of Medical School, Nanjing University, Nanjing, China; Center for Molecular Reproductive Medicine, Nanjing University, Nanjing, China; Center for Reproductive Medicine and Obstetrics and Gynecology, Nanjing Drum Tower Hospital, Affiliated Hospital of Medical School, Nanjing University, Nanjing, China; Center for Molecular Reproductive Medicine, Nanjing University, Nanjing, China; Center for Reproductive Medicine and Obstetrics and Gynecology, Nanjing Drum Tower Hospital, Affiliated Hospital of Medical School, Nanjing University, Nanjing, China; Center for Molecular Reproductive Medicine, Nanjing University, Nanjing, China; Center for Reproductive Medicine and Obstetrics and Gynecology, Nanjing Drum Tower Hospital, Affiliated Hospital of Medical School, Nanjing University, Nanjing, China; Center for Molecular Reproductive Medicine, Nanjing University, Nanjing, China; Fujian Provincial Key Laboratory of Reproductive Health Research, Department of Obstetrics and Gynecology, The First Affiliated Hospital of Xiamen University, School of Medicine, Xiamen University, Xiamen, China; Center for Reproductive Medicine and Obstetrics and Gynecology, Nanjing Drum Tower Hospital, Affiliated Hospital of Medical School, Nanjing University, Nanjing, China; Center for Molecular Reproductive Medicine, Nanjing University, Nanjing, China; State Key Laboratory of Reproductive Medicine and Offspring Health, Nanjing Medical University, Nanjing, China; Center for Reproductive Medicine and Obstetrics and Gynecology, Nanjing Drum Tower Hospital, Affiliated Hospital of Medical School, Nanjing University, Nanjing, China; Center for Molecular Reproductive Medicine, Nanjing University, Nanjing, China

**Keywords:** recurrent implantation failure, endometrial senescence, endometrial stromal cell, decidualization, CDC42, endometrial fibrosis

## Abstract

**STUDY QUESTION:**

Does the downregulation of cell division cycle 42 (CDC42) protein in endometrial stroma lead to endometrial senescence in patients with recurrent implantation failure (RIF), and what is the potential mechanism?

**SUMMARY ANSWER:**

CDC42 deficiency causes endometrial stromal senescence and decidualization defects, impairing uterine receptivity of RIF patients, via activation of Wnt signaling pathway.

**WHAT IS KNOWN ALREADY:**

Uterine aging is unique due to the cyclic remodeling and decidualization of endometrial tissue. Several transcriptomic studies have reported increased senescence in the endometrium in young patients with RIF. Our previous transcriptomic sequencing study discovered that endometrium from women with RIF showed downregulation of CDC42, which is an essential molecule affected by various senescence-related diseases.

**STUDY DESIGN, SIZE, DURATION:**

The endometrial samples of a total of 71 fertile control patients and 37 RIF patients were collected to verify the association between CDC42 expression and endometrial senescence of RIF patients. Primary endometrial stromal cells (EnSCs) were isolated from endometrial biopsies taken from patients without any endometrial complications and planning to undergo IVF, then subjected to adenovirus-mediated CDC42 knockdown and decidualization induction to explore the detailed mechanism by which CDC42 governs stromal senescence and decidualization. Wnt inhibitor XAV-939 was used to correct the endometrial senescence and decidualization defect.

**PARTICIPANTS/MATERIALS, SETTING, METHODS:**

Senescence was determined by cell cycle arrest markers (e.g. P16, P21, and P53), SASP molecules (e.g. IL6 and CXCL8), and SA-β-gal staining. Masson’s staining and Sirius Red staining were used to detect the endometrial fibrosis. Decidualization was evaluated by the mRNA expression and protein secretion of PRL and IGFBP1, F-actin immunostaining, and the BeWo spheroids ‘*in vitro* implantation’ model. Methods used to assess cell function included adenovirus transduction, RNA-sequencing, bioinformatic analysis, western blotting, RT-qPCR, ELISA, and immunofluorescence.

**MAIN RESULTS AND THE ROLE OF CHANCE:**

Here, we observed remarkably increased levels of stromal senescence and fibrosis, along with stromal CDC42 deficiency, in the endometrium of patients with RIF (*P* < 0.001). Knockdown of CDC42 effectively induced premature senescence in EnSCs, leading to aberrant accumulation of senescent EnSCs and collagen deposition during decidualization. CDC42 deficiency in EnSCs restrained the decidualization differentiation and receptivity to trophoblast cells. Transcriptomic analysis revealed Wnt signaling activation as a critical downstream alteration in CDC42-deficient EnSCs. Mechanistically, CDC42 interacted with AKT competitively to impede the binding of GSK3β to AKT. Knockdown of CDC42 increased AKT-mediated phosphorylation of GSK3β to inactivate the Axin-GSK3β destruction complex, leading to accumulation and nuclear translocation of β-catenin. Importantly, Wnt signaling inhibitors partially corrected the endometrial senescence caused by CDC42 deficiency, and improved both decidualization and trophoblast invasion.

**LARGE SCALE DATA:**

RNA-seq data sets generated in this study have been deposited at the NCBI database with BioProject accession number PRJNA1102745.

**LIMITATIONS, REASONS FOR CAUTION:**

The present study was based on *in vitro* cell cultures. Further studies involving CDC42-regulated endometrial senescence are needed in knockout mice model and human endometrial assembloids.

**WIDER IMPLICATIONS OF THE FINDINGS:**

In addition to uncovering endometrial senescence in RIF, our findings underscore the significance of CDC42 in modulating EnSC senescence to maintain the decidualization function, and suggest Wnt signaling inhibitors as potential therapeutic agents for alleviating endometrial senescence.

**STUDY FUNDING/COMPETING INTEREST(S):**

This work was supported by the National Natural Science Foundation of China [82271698 (R.J.), 82030040 (H.S.), 82288102 (H.W.), and 82371680 (G.Y.)]; the Natural Science Foundation of Jiangsu Province [BK20231117 (R.J.)]; and the Medical Science and Technology Development Foundation of Nanjing Department of Health [YKK23097 (Y.Z.)]. The authors declare no potential conflicts of interest.

## Introduction

In recent years, the issue of infertility among young women has become increasingly common (Farquhar *et al.*, 2019; Carson and Kallen, 2021), with a particular research focus on premature ovarian insufficiency (Armeni *et al.*, 2021; Shekari *et al.*, 2023), which is associated with premature senescence of the ovary. However, the role of senescence in the endometrium, another vital component of female fertility, has received much less attention. Senescence can be induced in different ways, including DNA damage accumulation, telomere shortening, oncogene activation, oxidative stress, and so on (Di Micco *et al.*, 2021). Accumulation of senescent cells within tissues has been proven to mediate dysfunction and the progression of various pathologies, such as chronic inflammation and fibrosis (Munoz-Espin and Serrano, 2014). The cyclic process of shedding and regeneration of the endometrium can impose significant proliferative pressure (Wang *et al.*, 2020), potentially exacerbating the aging processes within this tissue (Pathare *et al.*, 2023). Recent studies have reported a pro-inflammatory senescent decidual cell subpopulation under the acute stress response of decidualizing stimulation, which is quickly eliminated by uNK cells to assure the dominance of the decidual subpopulation during subsequent differentiation (Brighton *et al.*, 2017; Rawlings *et al.*, 2021). In addition, a pro-senescent decidual response in peri-implantation endometrium has been observed in patients with recurrent pregnancy loss (Lucas *et al.*, 2016, 2020). In view of the obvious importance of proper endometrial receptivity to female fertility, the senescence of the endometrium and its impacts on embryo implantation need to be further investigated.

Recurrent implantation failure (RIF) is a challenging issue in the field of reproductive medicine (Coughlan *et al.*, 2014; Franasiak *et al.*, 2021), and affects ∼15% of women undergoing *in vitro* fertilization and embryo transfer (IVF-ET) (Busnelli *et al.*, 2020). RIF patients fail to conceive even after multiple transfers of high-quality embryos (Coughlan *et al.*, 2014). Decreased endometrial receptivity accounts for two-thirds of the implantation failures in RIF patients (Craciunas *et al.*, 2019). Decidualization is the key step in endometrial receptivity establishment, which is characterized by the functional differentiation of endometrial stromal cells (EnSCs) (Lessey and Young, 2019). Some studies have presented the relationship between EnSC senescence and decidualization (Kusama *et al.*, 2014; Liao *et al.*, 2015). The short-term presence of senescent EnSCs appeared to be essential for the proper onset of decidualization (Ochiai *et al.*, 2019a,b), but an inappropriate abundance of senescent EnSCs, together with an altered EnSC secretome, preceded implantation failure (Deryabin *et al.*, 2020; Deryabin and Borodkina, 2022). In particular, decidualization defects and implantation failure have been linked to the enhanced level of premature senescence of EnSCs during the proliferation phase (Tomari *et al.*, 2020; Deryabin and Borodkina, 2022). Recently, several studies of transcriptomic sequencing analyses have reported increased senescence of the endometrium in young patients with RIF (Chen *et al.*, 2022; Zhao *et al.*, 2022), suggesting that endometrial senescence might be the underlying cause of decreased endometrial receptivity in these individuals. Nevertheless, experimental studies validating this hypothesis and exploring its molecular mechanisms are still lacking.

Cell division cycle 42 (CDC42), an important molecule involved in the regulation of multiple physiological functions (Farhan and Hsu, 2016; Mei *et al.*, 2022), was found to be downregulated in the endometrium of patients with RIF in our previous transcriptomic sequencing study (Zhou *et al.*, 2019). Moreover, numerous studies have highlighted a significant correlation between changes in CDC42 expression and senescence-related diseases, in the central nervous system (Ying *et al.*, 2022), cardiovascular system (Flentje *et al.*, 2019), and osteoarticular system (Yuan *et al.*, 2021). However, the specific role of CDC42 in endometrial function, whether it participates in the regulation of endometrial senescence, and its association with abnormal receptivity of RIF patients remains unknown. In this study, we collected endometrial samples and primary EnSCs to reveal that deficient CDC42 led to endometrial senescence and inhibited decidualization. Our findings provide a novel mechanistic insight into how CDC42 governs EnSC senescence, and may have implications for the treatment of endometrium-associated infertility.

## Materials and methods

### Patients and sample collection

Endometrial biopsy samples were obtained from women undergoing IVF-ET treatment at the Reproductive Medicine Center of Nanjing Drum Tower Hospital. Prior to sample collection, informed consent was obtained from all patients. The study included women aged between 20 and 35 years, who had regular cyclic menses (25–32 days) and had not received any hormone therapy for a minimum of 3 months before tissue collection. Fertile control (CTR) patients were defined as women who underwent IVF-ET treatment due to male infertility factor and became pregnant after the first embryo transfer. RIF patients were defined as individuals who failed to achieve clinical pregnancy despite the transfer of at least four high-quality embryos over a minimum of three fresh or frozen cycles (Coughlan *et al.*, 2014). Exclusion criteria included: a known uterine abnormality (e.g. uterine congenital malformation, untreated uterine septum, submucous myoma, endometrial polyps, or intrauterine adhesions); a thin endometrium (<6 mm); hydrosalpinx diagnosed by hysterosalpingogram; endometritis diagnosed by hysteroscopy; endometriosis or adenomyosis diagnosed by transvaginal ultrasonography; known autoimmune diseases, currently taking corticosteroids or confounding immunosuppression medications; or abnormal results on parental karyotyping. Mid-secretory endometria were sampled between the 20th and 24th days of the cycle, about 5–7 days after ovulation suggested by ultrasound. Proliferative endometria were sampled between the 7th and 10th days of the cycle, with a typical three-layer ultrasound pattern. The endometrial biopsies were snap-frozen in liquid nitrogen for RNA or protein extraction, or placed in 10% neutral formalin for paraffin embedding, or collected in DMEM-F12 media for isolation of primary EnSCs. The Institutional Review Board of Nanjing Drum Tower Hospital granted approval for the use of human tissues (2013-081-05). The information regarding the patients recruited is presented in Supplementary Table S1.

### Isolation of primary human EnSCs

The primary human EnSCs were isolated following a previously described protocol (Wang *et al.*, 2021). Briefly, freshly collected mid-secretory endometrial specimens were finely minced and subjected to enzymatic digestion using 0.1% collagenase I (Sigma) at 37°C for 30 min. The dissociated stromal cells were then separated from the intact glandular structures by filtering the enzymatically digested tissue through a 40-μm sieve. Subsequently, the flow-through cells were centrifuged at 1000 × *g* for 5 min. The resulting cell pellet was resuspended and cultured in DMEM/F12 (Corning) supplemented with 10% fetal bovine serum (FBS) (Gibco), 100 IU/ml penicillin, and 100 μg/ml streptomycin (HyColne) in a humidified incubator at 37°C with 5% CO_2_. For this study, cells within passages 2 through 4 were utilized.

### EnSC culture and decidualization differentiation induction

The primary human EnSCs were cultured as above. The immortalized human EnSCs (T-EnSCs) used in this study were commercially obtained from ATCC (No. CRL-4003). Decidualization induction of primary EnSCs or T-EnSCs was achieved by administering 0.5 mM 8-bromoadenosine 3′,5′-cyclic monophosphate (8-Br-cAMP) (Sigma) and 1 μM medroxyprogesterone acetate (MPA) (Sigma) in phenol red-free DMEM/F12 medium (Corning), supplemented with 2.5% charcoal-stripped FBS (Gibco). Primary EnSCs or T-EnSCs underwent a preliminary 2-day treatment with 100 moi Ad-GFP or Ad-shCDC42 to knockdown the expression of CDC42, or 0.1% DMSO or 10 μM ML141 (MCE, HY-12755) to inhibit the GTPase activity of CDC42, followed by a 3-day exposure to both 8Br-cAMP and MPA, and were then harvested for downstream analysis. For inhibitor studies, 2 μM MK2206 (MCE, HY-108232), 25 μM LY294002 (MCE, HY-10108), or 20 μM XAV-939 (MCE, HY-15147) were added to the medium 24 h after adenovirus treatment.

### Enzyme-linked immunosorbent assay

Cell culture medium supernatant was harvested and centrifuged to remove cell debris. Commercially available ELISA kits were utilized to measure the level of secreted protein (PRL: Elabscience, No. E-EL-H0141; IGFBP1: Elabscience, No. E-EL-H0442; IL6: Elabscience, No. E-EL-H6156; IL8: MULTI SCIENCES, No. EK108-96; CLU: Elabscience, No. E-EL-H0038; sST2: Elabscience, No. E-EL-H6082) in collected supernatants according to the manufacturer’s instructions.

### BeWo spheroids ‘*in vitro* implantation’ model

The BeWo cell line was purchased from ATCC (No. CCL-98). Cells were cultured in DMEM/F12 (Corning) supplemented with 10% FBS (Clark), 100 IU/ml of penicillin, and 100 μg/ml of streptomycin (HyColne) in a humidified incubator at 37°C with 5% CO_2_. The ‘*in vitro* implantation’ model implies the estimation of the spreading area of spheroids formed from BeWo cells (Weimar *et al.*, 2013). BeWo spheroids were formed by transferring single-cell suspension of BeWo cells into dishes coated with Poly-2-hydroxyethyl methacrylate (Poly-HEMA, Sigma) and cultured at 37°C with 5% CO_2_ for 24 h. In our study, T-EnSCs treated with indicated adenovirus or inhibitor were subject to decidualization induction for 2 days. Then, the BeWo spheroids were seeded onto the monolayers of decidualized T-EnSCs and co-cultured for 4 days, with images captured per 24 h. Quantitative analysis of the spreading area was conducted with the application of Adobe Photoshop (version 2022) by calculating the ratio of invaded area to total area of the whole images.

### Adenovirus construction

An adenovirus harboring shRNA targeted to CDC42 (Ad-shCDC42-GFP) was generated as previously described (Cai *et al.*, 2022), with the following shCDC42 sequence: GACTCAAATTGATCTCAGA. Briefly, shCDC42 sequence was cloned into pacAd5 U6-GFP plasmid vector, followed by adenovirus construction. The adenoviruses were propagated in HEK293A cells and purified via CsCl banding, followed by dialysis against 20 mmol/l Tris-buffered saline with 10% glycerol. Titration was performed on HEK293A cells using Adeno-X Rapid Titer kit (Clontech) according to the manufacturer’s instructions. Ad-GFP without specific shRNA was used as the control virus. An adenovirus harboring the coding sequence of CDC42 (Ad-CDC42-flag, NCBI Reference Sequence: NM_001039802.2) was generated as previously described (Zhou *et al.*, 2019). The adenovirus-bearing LacZ vector (Ad-LacZ) was obtained from Clontech and was used as a control in the adenovirus-mediated CDC42 overexpression experiments. The adenoviruses were used at the concentration of 100 moi unless otherwise stated.

### Immunohistochemistry

Tissue specimens were fixed in 10% neutral formalin and subsequently embedded in paraffin. Sections were deparaffinized, rehydrated, and subjected to antigen retrieval using a high-pressure method. To quench endogenous peroxidase activity, sections were treated with 3% H_2_O_2_ for 10 min. Non-specific binding was blocked by incubating with 10% normal goat serum for 1 h at room temperature. The sections were then incubated with primary antibodies (as listed in Supplementary Table S2) in a humidified chamber at 4°C overnight, followed by room-temperature incubation with HRP conjugated secondary antibodies (BOSTER) for 1 h. Subsequent staining was performed with 3,3′-diaminobenzidine and counterstaining was done with hematoxylin. Digital images of the sections were acquired using a Leica DM 2000 microscope. The relative protein expression levels in the endometrial samples were quantitatively assessed by measuring the integrated optical density (IOD) of the digital images at 200× magnification using the Image-Pro Plus System 6.0 as described previously (Cai *et al.*, 2023). The relative protein expression levels of P16 and P21 were also measured by the average number of P16^+^ or P21^+^ cells in three randomly selected 200× magnification field for each CTR or RIF patient.

### Immunofluorescence

EnSCs were fixed using 4% paraformaldehyde for 15 min at room temperature, followed by permeabilization with 0.1% Triton X-100 in PBS for 5 min at room temperature and then blocked using 5% bovine serum albumin (BSA) in PBS for 1 h at room temperature. The cells were then incubated with primary antibodies (listed in Supplementary Table S2) overnight at 4°C. Fluorescence-conjugated secondary antibodies were employed to detect the primary antibody binding (Thermo A-21207 and A-11008). Nuclear staining was achieved with 4′,6-diamidino-2-phenylindole dihydrochloride (DAPI) (Servicebio) for 10 min at room temperature. Subsequently, images were captured with fluorescence microscopy and processed using LAS X software.

### Masson’s staining and Sirius Red staining

Masson’s staining was conducted using Masson Tri-color dyeing solution (Leagene, DC0033) following the manufacturer’s instructions. Sirius Red staining was conducted using Picrosirius Red Staining Solution (PH1098, PHYGENE). After dewaxing, the sections were stained with Sirius Red staining solution at room temperature for 1 h. Subsequently, dehydration was performed using ethanol and xylene in a sequential manner, followed by mounting the slides.

### SA-β-galactosidase staining

To evaluate senescence in primary EnSCs and T-EnSCs, we employed the Senescence β-Galactosidase Staining Kit (Beyotime Institute of Biotechnology, C0602). The cells were first washed with PBS and then fixed with the fixative provided in the kit for 15 min at room temperature. After removal of the fixative and washing with PBS, EnSCs were incubated with a staining solution containing X-gal in a pH 6.0 buffer. The staining process was carried out for 16 h at 37°C in a non-CO_2_ incubator. Bright-field micrographs of three randomly selected fields from each group were captured. Quantitative analysis of senescence was conducted using Image-Pro Plus software to measure the IOD of the SA-β-Gal-positive cells.

### Co-immunoprecipitation

The T-EnSCs were treated with indicated plasmids or adenoviruses for 72 h, followed by lysis in a buffer containing protease inhibitors (Roche) and phosphatase inhibitors (Sigma). The cell lysates underwent pre-clearance using protein A/G beads (Abmart, No. A10001M) at 4°C for 2 h. Subsequently, purified cell extracts were incubated overnight at 4°C with either 5 μg of the primary antibody or isotype IgG. Antibodies used in co-immunoprecipitation are listed in Supplementary Table S2. The antibody-conjugated cell extracts were added to Protein A/G beads and incubated at 4°C for 4 h. For immunoprecipitation of exogenous proteins, the purified cell extracts were added to Flag-beads (Sigma, No. F1804) or HA-beads (AlpaLifeBio, No. KTSM1305) and incubated overnight at 4°C. Finally, the proteins bound to the beads were released by adding 2× Laemmli sample buffer and subjected to 95°C for 5 min. The separated and collected proteins were analyzed using western blot.

### Western blot

Protein extraction was carried out in accordance with the methods previously outlined (Cai *et al.*, 2022). Equivalent quantities of protein were separated by SDS-polyacrylamide gel electrophoresis and subsequently transferred onto polyvinylidene fluoride membranes (Millipore). The membranes were blocked with 5% skim milk for 1 h at room temperature and then incubated overnight at 4°C with primary antibodies (listed in Supplementary Table S2), followed by incubation with HRP-conjugated secondary antibody for 1 h at room temperature. Detection was carried out utilizing an enhanced chemiluminescence kit (Millipore, WBKLS0500). The expression level of the target protein was normalized to that of GAPDH in the corresponding sample, enabling accurate quantification of relative abundance. Signal intensities were quantified using ImageJ software’s densitometric analysis.

### Quantitative real-time PCR

Cellular lysis was achieved using TRIzol reagent (Ambion), and total RNA was subsequently isolated in accordance with the manufacturer’s standard protocol. A maximum of 2 µg of total RNA was reverse transcribed in a 20-µl reaction volume to synthesize cDNA utilizing the 5× All-In-One RT Master Mix (abm, G492). Quantitative real-time PCR was then conducted employing 2× SYBR dye (Vazyme), with the amplification signals detected on an Analytik Jena qPCR system. For normalization purposes, the constitutively expressed 18S rRNA gene served as an internal reference. The sequences of the specific primers utilized are delineated in Supplementary Table S3.

### RNA-seq and bioinformatic analysis

Primary EnSCs were subjected to 100 moi adenovirus treatment for 48 h, followed by decidualization induction for an additional 72 h. Total RNA was extracted using TRIzol reagent (Ambion). Subsequently, RNA-sequencing (RNA-seq) and data analysis were conducted by Majorbio Co., Ltd (Shanghai, China). The obtained clean reads were aligned to the human reference genome using HISAT2. Bioinformatic analysis was conducted using R software (version 4.2.2). Principal components analysis (PCA) was performed using the built-in R function of princomp. To identify differentially expressed genes (DEGs), the DESeq2 R package was employed (version 1.36.0), with a significance threshold set at *P*-value <0.05 and |log2-foldChange|>1. Genetic ontology (GO), Kyoto Encyclopedia of Genes and Genomes (KEGG), and Gene Set Enrichment (GSEA) analyses were performed using clusterProfiler R package (version 4.8.1). Heatmap analysis was performed using the built-in R function of pheatmap.

### Statistical analysis

Each experiment was conducted for a minimum of three separate replicates to ensure reproducibility. Statistical evaluations were carried out utilizing GraphPad Prism software (version 9). The data are presented as the mean±SEM of the biological replicates. Comparative analyses of the mean expression values between two distinct treatment groups were executed using a 2-tailed Student’s *t*-test. For multiple-group comparisons, one-way ANOVA was employed. Two-way ANOVA was employed when both the time of decidualization and different treatments had impacts on the results.

## Results

### Cellular senescence in the stroma of RIF endometrium

**Figure 1.**
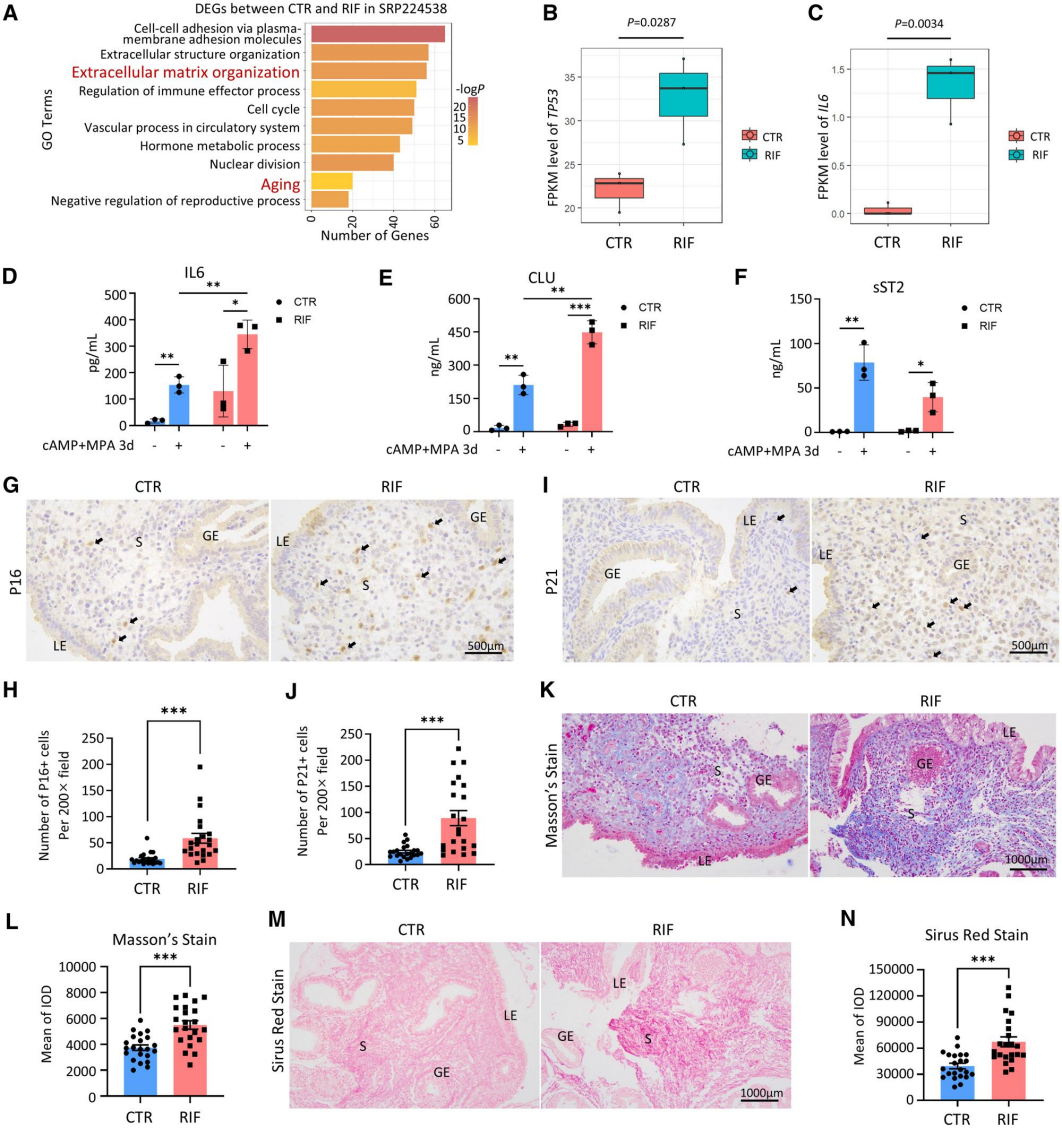
**Cellular senescence in the stroma of recurrent implantation (RIF) failure endometrium.** (**A**) Gene ontology (GO) biological process enrichment of the differentially expressed genes (DEGs) between fertile control (CTR) patients and patients with RIF in SRP224538. (**B**) FPKM level of *TP53* in CTR patients and RIF patients in SRP224538. (**C**) FPKM level of *IL6* in CTR patients and RIF patients in SRP224538. (**D**–**F**) Levels of IL6, CLU, and sST2 secretion in primary endometrial stromal cell supernatant from CTR and RIF patients before and after 72 h of 8Br-cAMP+MPA treatment. (**G** and **H**) Representative images of immunohistochemical (IHC) staining of P16 in mid-secretory endometrium of CTR patients (n = 22) versus RIF patients (n = 22). Black arrows indicate typical P16^+^ cells. Quantitative analysis by number of P16^+^ cells per 200× field for P16 staining. (**I** and **J**) Representative images of IHC staining of P21 in mid-secretory endometrium of CTR patients (n = 22) versus RIF patients (n = 22). Black arrows indicate typical P21^+^ cells. Quantitative analysis by number of P21^+^ cells per 200× field for P21 staining. (**K** and **L**) Representative images of Masson’s staining in mid-secretory endometrium of CTR patients (n = 22) versus RIF patients (n = 22). Quantitative analysis of integrated optical density (IOD) for Masson’s staining. (**M** and **N**) Representative images of Sirus Red staining in mid-secretory endometrium of CTR patients (n = 22) versus RIF patients (n = 22). Quantitative analysis of IOD for Sirus Red staining. LE, luminal epithelium; GE, glandular epithelium; S, stroma. Mean±SEM. ***P < 0.001. Student’s *t*-test.

To reveal whether endometrial senescence occurs in the endometrium of RIF patients, we re-analyzed the transcriptomic data in our previous study (SRP224538) (Zhou *et al.*, 2019). GO enrichment analysis of DEGs between fertile control (CTR) and RIF groups included pathways associated with ‘aging’ and ‘extracellular matrix’ (Fig. 1A). In addition, *TP53*, a classic marker gene of senescence, and *IL6*, a molecule of the senescence-associated secretory phenotype (SASP), were both significantly increased in the endometrium of RIF patients (Fig. 1B and C). The increased secretion of IL6 was verified by ELISA of the primary EnSC supernatant obtained from mid-secretory CTR and RIF endometria, and the upregulation in IL6 persisted after 72 h of 8Br-cAMP+MPA treatment (Fig. 1D). In addition, increased Clusterin (CLU) secretion and decreased sST2 (encoded by *IL1RL1*) secretion were found in RIF EnSCs after decidualization stimulation compared with CTR EnSCs (Fig. 1E and F), indicating an increased level of senescence and decreased level of decidualization, respectively (Lucas *et al.*, 2020). Immunohistochemical staining revealed a significantly increased number of P16 and P21 positive cells in the endometrium of RIF patients, primarily in the stromal cells with minimal changes in the epithelial cells (n = 22 for each group) (Fig. 1G–J, Supplementary Fig. S1A and B), indicating senescence appears predominantly in the EnSCs of RIF patients. Masson’s staining and Sirius Red staining further showed increased collagen deposition in the stroma of RIF patients (n = 22 for each group) (Fig. 1K–N). Senescence contributes to fibrosis in multiple organs (Pathare *et al.*, 2023), so we conducted a linear regression analysis of the correlation between P16/P21 immunohistochemical staining and Masson’s staining/Sirius Red staining, and discovered that endometrial fibrosis was in a positive correlation with endometrial senescence (Supplementary Fig. S1C–F). The above transcriptomic and histopathologic analysis indicates that the RIF patients exhibit remarkable cell senescence in the endometrial stroma.

### CDC42 knockdown accelerates senescence of differentiated EnSCs

**Figure 2.**
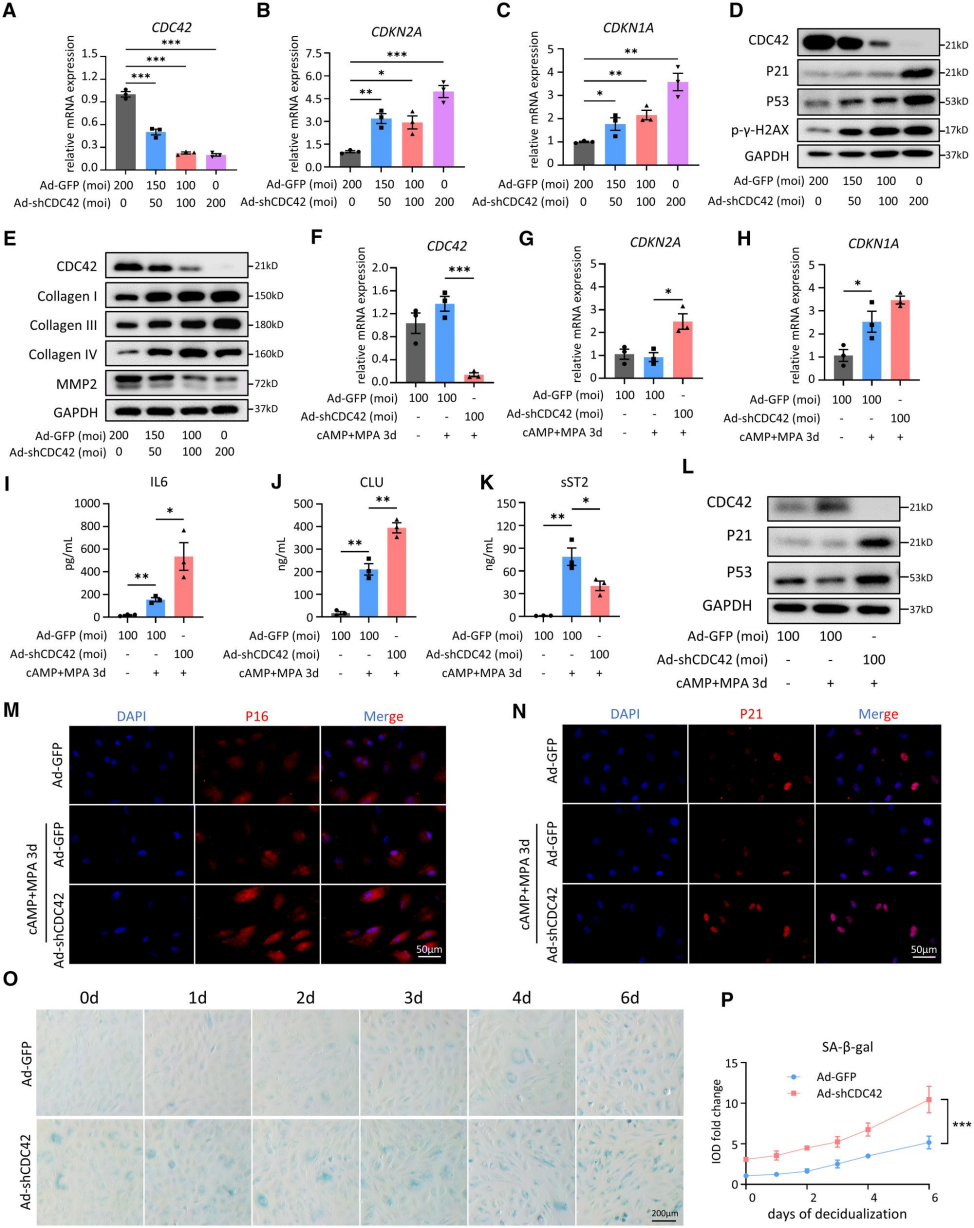
**CDC42 knockdown accelerates senescence of differentiated endometrial stromal cells (EnSCs).** (**A**–**C**) Expression of *CDC42*, *CDKN2A*, and *CDKN1A* mRNA levels in primary EnSCs after 72 h of adenovirus treatment. (**D**) Expression of CDC42, P21, P53, and p-γ-H2AX protein levels in primary EnSCs after 72 h of adenovirus treatment. (**E**) Expressions of collagen I, collagen III, collagen IV, and MMP2 protein levels in primary EnSC after 72 h of adenovirus treatment. (**F**–**H**) Expressions of *CDC42*, *CDKN2A*, and *CDKN1A* mRNA levels in primary EnSC transfected with Ad-GFP or Ad-shCDC42-GFP following with 72 h of 8Br-cAMP+MPA treatment. (**I**–**K**) Level of IL6, CLU, and sST2 secretion in primary EnSCs transfected with Ad-GFP or Ad-shCDC42-GFP following with 72 h of 8Br-cAMP+MPA treatment. (**L**) Expression of CDC42, P21, and P53 protein levels in primary EnSCs transfected with Ad-GFP or Ad-shCDC42-GFP following with 72 h of 8Br-cAMP+MPA treatment. (**M** and **N**) Representative images of P16 and P21 immunofluorescence staining in primary EnSCs transfected with Ad-GFP or Ad-shCDC42-GFP following with 72 h of 8Br-cAMP+MPA treatment. (**O** and **P**) SA-β-gal staining of primary EnSCs after 48 h of Ad-GFP or Ad-shCDC42-GFP adenovirus treatment and then different days of 8Br-cAMP+MPA treatment. Quantitative analysis of integrated optical density for SA-β-gal staining, with value of the Ad-GFP group at 0 h set as 1. Mean ± SEM. *P < 0.05, **P < 0.01, ***P<0.001. ANOVA with Tukey’s multiple comparisons test. Two-way ANOVA with the Bonferroni multiple comparisons test in (P).

In our previous transcriptomic sequencing study, CDC42 was found to be downregulated in the endometrium of RIF patients (Zhou *et al.*, 2019). To investigate the potential role of CDC42 in cellular senescence of endometrial stroma, primary EnSCs obtained from CTR patients were treated with adenovirus loaded with sh-CDC42 (Ad-shCDC42). The CDC42-knockdown adenovirus efficiently reduced *CDC42* expression in a dose-dependent manner (Fig. 2A). CDC42-knockdown increased the expression of classic senescence markers such as *CDKN2A*, *CDKN1A* (Fig. 2B and C), and *TP53* (Supplementary Fig. S2A), as well as the expression of SASP molecules such as *IL6*, *IL1A*, *IL1B*, *TGFB1*, and *CXCL8* (Supplementary Fig. S2B–F). Western-blot analysis also indicated that CDC42-knockdown increased the expression of P21, P53, and the DNA damage-related molecule p-γ-H2AX in a dose-dependent manner (Fig. 2D). In addition, the expression levels of collagen I, collagen III, and collagen IV were increased, but collagenase MMP2 level was decreased after CDC42 knockdown (Fig. 2E), indicating that CDC42 deficiency results in senescence-related fibrosis in endometrial stroma. Since endometrial stroma experiences cyclical decidualization, it is necessary to further investigate the role of CDC42 deficiency in EnSC senescence during decidual differentiation. Primary EnSCs treated with Ad-shCDC42 were subjected to decidualization induction of 8Br-cAMP+MPA for 3 days. RT-qPCR assays showed that CDC42 knockdown still upregulated *CDKN2A*, *CDKN1A*, and *IL6* mRNA levels in differentiated EnSCs (Fig. 2F–H, Supplementary Fig. S2G), albeit *CXCL8* was downregulated in CDC42-knockdown differentiated EnSCs (Supplementary Fig. S2H). ELISA of supernatant from the EnSCs further confirmed the increased secretion of IL6 and decreased secretion of IL8 (Fig. 2I, Supplementary Fig. S2I), as well as upregulated secretion of CLU and downregulated secretion of sST2 (Fig. 2J and K). Western blot suggested that CDC42 deficiency led to increased expression of P21 and P53 in differentiated EnSCs (Fig. 2L). Immunofluorescence staining further demonstrated that CDC42 knockdown increased the number of P16 and P21 positive EnSCs during decidualization (Fig. 2M and N, Supplementary Fig. S2J). To elucidate in detail the role of CDC42 on EnSC senescence during decidualization, we conducted continuous SA-β-gal staining for 6 days. Primary EnSCs began to present a mild senescence phenotype upon 3 days of decidualization, which was further exacerbated after 6 days of decidualization. Importantly, CDC42 deficiency led to a premature senescence before differentiation induction (equivalent to that of the control group stimulated for 4 days), and accelerated accumulation of SA-β-gal positive EnSCs from 4 to 6 days (Fig. 2O and P). Together, these results indicated that deficiency of CDC42 induced premature senescence of EnSCs, leading to aberrant accumulation of senescent EnSCs during decidualization.

### Decidualization-associated CDC42 upregulation is suppressed in RIF endometria

**Figure 3.**
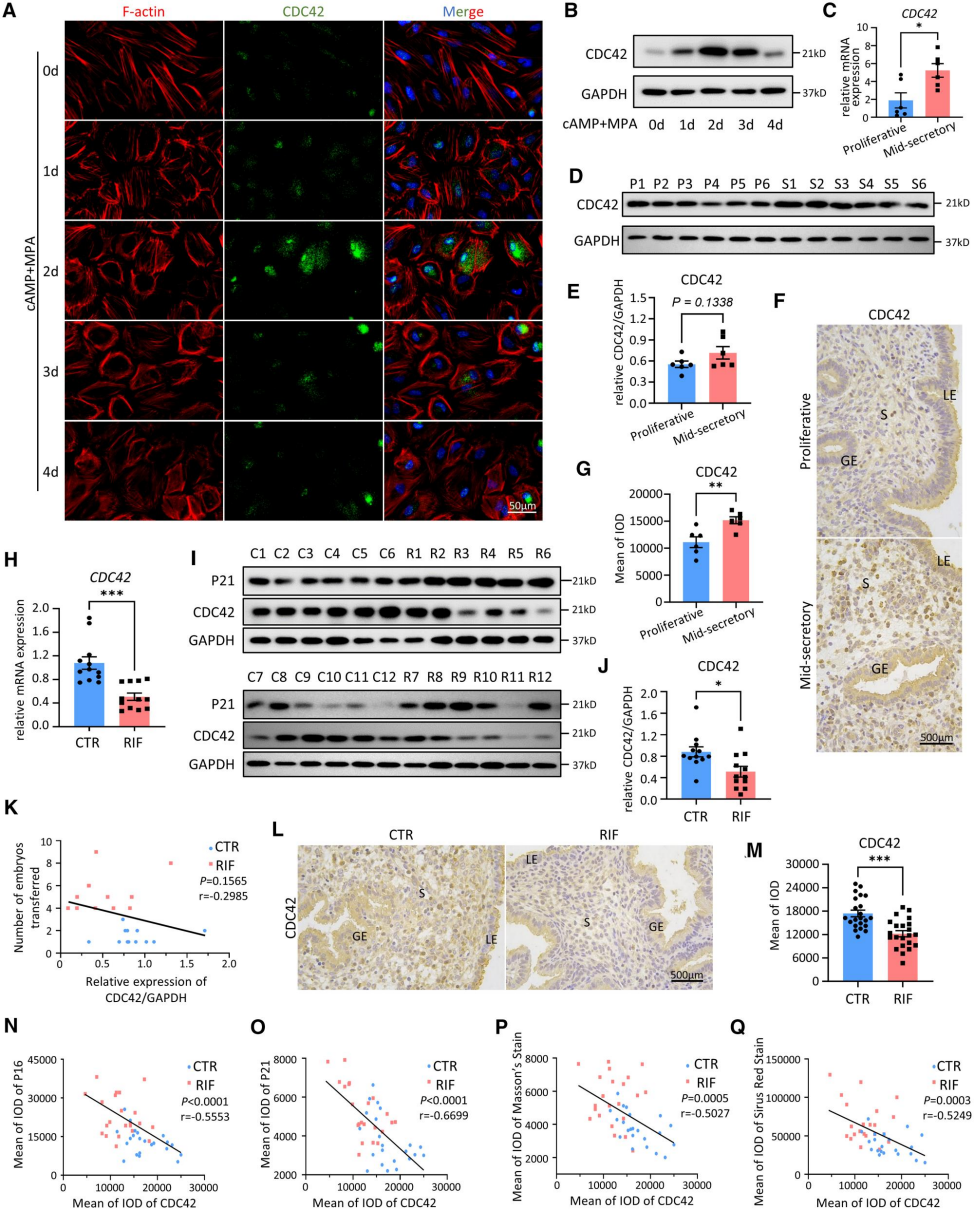
**Decidualization-associated CDC42 upregulation is suppressed in recurrent implantation failure (RIF) endometria.** (**A**) Representative images of F-actin and CDC42 immunofluorescence staining in endometrial stromal cells (EnSCs) at different time points after 8Br-cAMP+MPA treatment. (**B**) Expression of protein levels of CDC42 in EnSCs at different time points after 8Br-cAMP+MPA treatment. (**C**) Comparison of *CDC42* mRNA levels between proliferative endometrium of control (CTR) patients (n = 6) and mid-secretory endometrium of CTR patients (n = 6) using qRT-PCR. (**D** and **E**) Western-blot analysis of CDC42 protein level in proliferative endometrium of CTR patients (n = 6) and mid-secretory endometrium of CTR patients (n = 6). Quantitative analysis of protein level of CDC42 with GAPDH as the control. Each Lane indicates a single patient. P, Proliferative Control. S, Mid-secretory CTR. (**F** and **G**) Immunohistochemical (IHC) staining of CDC42 in proliferative endometrium of CTR patients (n = 6) versus mid-secretory endometrium of CTR patients (n = 6). Quantitative analysis of integrated optical density (IOD) for CDC42 staining. (**H**) Comparison of endometrial *CDC42* mRNA levels between CTR patients (n = 12) and RIF patients (n = 12) using qRT-PCR. (**I** and **J**) Western-blot analysis of CDC42 and P21 protein levels in the mid-secretory endometrium from CTR patients (C, n = 12) and RIF patients (R, n = 12). Quantitative analysis of protein level of CDC42 with GAPDH as the control. Each Lane indicates a single patient. C, Mid-secretory Control. R, Mid-secretory RIF. (**K**) Linear regression analysis of the correlation between relative expression of CDC42/GAPDH and the number of embryos transferred in CTR patients (n = 22) and RIF patients (n = 22). (**L** and **M**) Representative images of IHC staining of CDC42 in mid-secretory endometrium of CTR patients (n = 22) versus RIF patients (n = 22). Quantitative analysis of IOD for CDC42 staining. (**N**–**Q**) Linear regression analysis of the correlation between IOD of IHC staining for (**M**) CDC42 and P16, (**N**) CDC42 and P21, (**O**) CDC42 and Masson’s staining, and (**P**) CDC42 and Sirus Red staining in CTR patients (n = 22) and RIF patients (n = 22). LE, luminal epithelium; GE, glandular epithelium; S, stroma. Mean±SEM. *P<0.05, **P<0.01, ***P<0.001. Student’s *t*-test.

Next, we explored the physiological and pathological change of CDC42 in the endometrium. A major characteristic of EnSC decidualization is the cytoskeleton remodeling process, in which EnSCs transform from spindle-shaped fibroblast-like cells to polygonal epithelial-like cells (Owusu-Akyaw *et al.*, 2019). We found that CDC42 expression peaked on Day 2 of decidualization, when EnSCs completed cytoskeletal remodeling, and returned to baseline by Day 4 (Fig. 3A and B). Therefore, CDC42 probably favors the onset of decidualization, while downregulation of CDC42 is associated with senescence in the late stages of decidualization. We next examined the dynamic expression pattern of endometrial CDC42 in normal menstrual cycles, as well as the difference in its expression between CTR and RIF endometria. Compared with proliferative endometrium, the mid-secretory endometrium displayed a markedly elevated CDC42 mRNA and protein levels (Fig. 3C–E). Immunohistochemical staining further showed a distinct upregulation of CDC42 in the stromal cells of mid-secretory endometrium (Fig. 3F and G). However, RT-qPCR and western-blot analyses revealed that CDC42 levels were decreased in RIF patients compared with CTR women, and found a negative correlation between CDC42 and P21 protein expression (Fig. 3H–J). It was noticed that not all RIF patients exhibited low levels of CDC42 and high levels of P21 (e.g. R1 and R11). Albeit women with low CDC42 expression received more embryos transferred, there was no significant negative correlation between them (Fig. 3K). These data indicated the heterogeneity of RIF patients. Immunohistochemical staining of CDC42 verified that, compared with fertile controls, RIF patients exhibited pronounced reduction of CDC42 expression in EnSCs (n = 22 for each group) (Fig. 3L and N). We further found a significant negative correlation between the expression levels of P16 with CDC42 (*r* = −0.5553, n = 44) and P21 with CDC42 (*r* = −0.6699, n = 44) (Fig. 3N and O). In addition, the degree of endometrial collagen deposition was also negatively correlated with CDC42 expression levels (*r* = −0.5027, n = 44 for Masson’s staining and *r* = −0.5249, n = 44 for Sirius Red staining) (Fig. 3P and Q). All of the above data suggested that reduced CDC42 expression contributes to endometrial senescence in RIF, especially in the uterine stroma.

### Senescence in CDC42-knockdown stromal cells inhibits decidualization and trophoblast invasion

**Figure 4.**
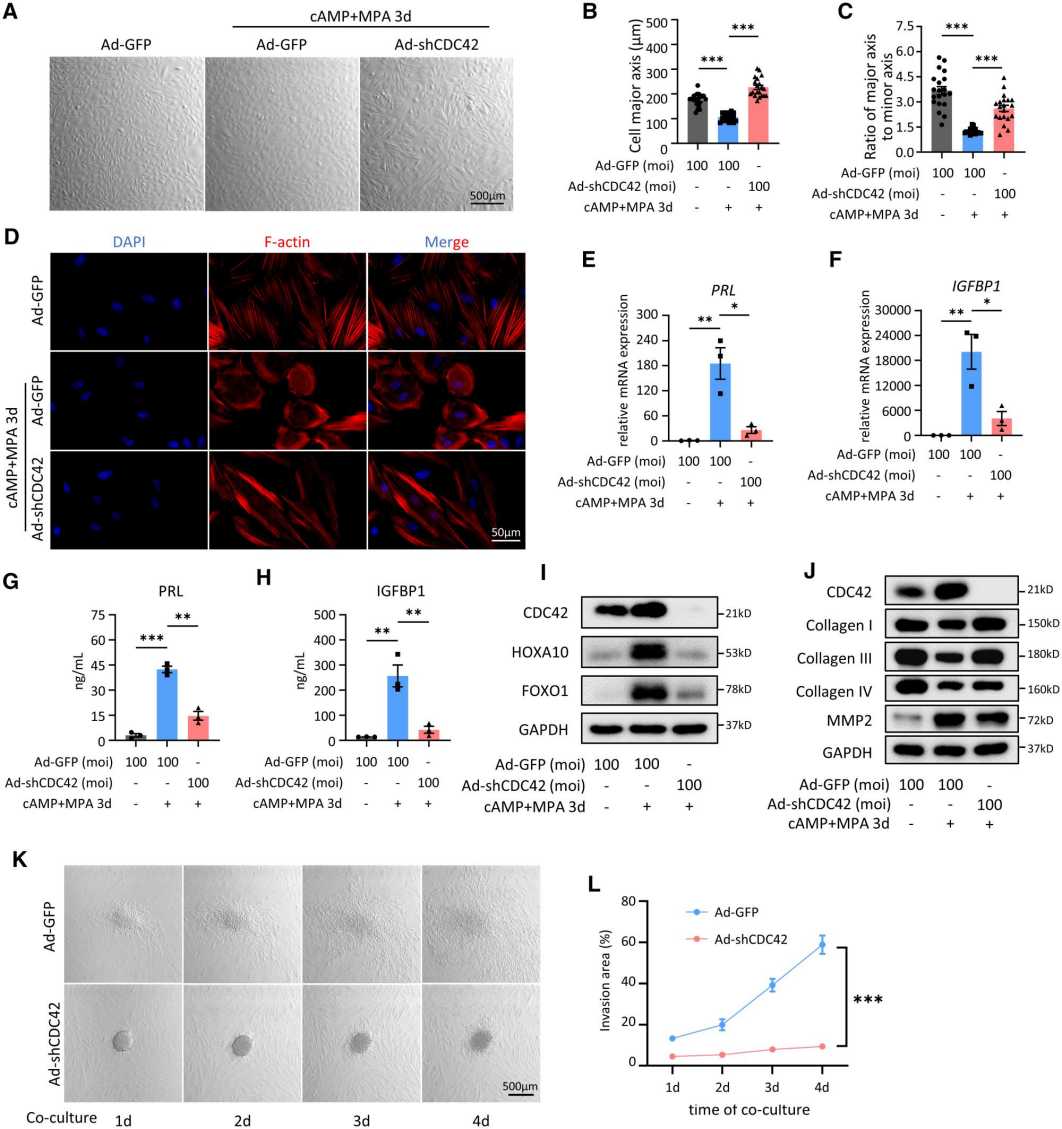
**Senescence in CDC42-knockdown stromal cells inhibits decidualization and trophoblast invasion.** (**A**–**C**) Representative bright filed images of primary endometrial stromal cells (EnSCs) transfected with Ad-GFP (control) or Ad-shCDC42-GFP (CDC42 knockdown) adenoviruses following with 72 h of 8Br-cAMP+MPA treatment. Quantitative analysis of cell major axis and the ratio of cell major axis to minor axis. (**D**) Representative images of F-actin immunofluorescence staining in primary EnSCs transfected with Ad-GFP or Ad-shCDC42-GFP following with 72 h of 8Br-cAMP+MPA treatment. (**E** and **F**) Expression of *PRL* and *IGFBP1* mRNA levels in primary EnSCs transfected with Ad-GFP or Ad-shCDC42-GFP following with 72 h of 8Br-cAMP+MPA treatment. (**G** and **H**) Level of PRL and IGFBP1 secretion in primary EnSCs transfected with Ad-GFP or Ad-shCDC42-GFP following with 72 h of 8Br-cAMP+MPA treatment. (**I**) Expression of HOXA10 and FOXO1 protein levels in primary EnSCs transfected with Ad-GFP or Ad-shCDC42-GFP following with 72 h of 8Br-cAMP+MPA treatment. (**J**) Expression of collagen I, collagen III, collagen IV, and MMP2 protein levels in primary EnSCs transfected with Ad-GFP or Ad-shCDC42-GFP following with 72 h of 8Br-cAMP+MPA treatment. (**K** and **L**) Representative bright filed images of different days after Bewo spheroids invaded the immortalized EnSCs (T-EnSCs) transfected with Ad-GFP or Ad-shCDC42-GFP following with 48 h of 8Br-cAMP+MPA treatment. Quantitative analysis of invasion area of Bewo spheroids. Mean±SEM. *P<0.05, **P<0.01, ***P<0.001. ANOVA with Tukey’s multiple comparisons test. Two-way ANOVA with the Bonferroni multiple comparisons test in (L).

Next, we detected the impact of CDC42 deficiency on the decidual differentiation of EnSCs. We noticed that the control adenovirus-treated primary EnSCs showed overt transformation from spindle-shaped fibroblast-like cells (ratio of major axis to minor axis = 3.67) to polygonal epithelial-like cells (ratio of major axis to minor axis = 1.25) after 3 days of 8Br-cAMP and MPA stimulation, while EnSCs with CDC42 knockdown still appeared as spindle-shaped fibroblast-like cells (ratio of major axis to minor axis = 2.61) with enlarged cell size (Fig. 4A–C). Immunofluorescence staining of F-actin further confirmed that CDC42 knockdown hindered the decidual transformation of primary EnSCs but resulted in an increased cell size (Fig. 4D). CDC42 knockdown impaired the upregulated mRNA expression and protein secretion of decidualization markers PRL and IGFBP1 (Fig. 4E–H). Western-blot analysis showed that CDC42 deficiency hindered the upregulation of HOXA10 and FOXO1 (Fig. 4I), two key regulators of decidualization progression (Gellersen and Brosens, 2014; Jiang *et al.*, 2017; Adiguzel and Celik-Ozenci, 2021). Upon decidualization, differentiated EnSCs favor the invasion of embryonic trophoblast by remodeling extracellular matrix (Anacker *et al.*, 2011; Winkler *et al.*, 2024). However, CDC42 knockdown impeded the reduction of collagen I and collagen III during decidualization (Fig. 4J), which may limit trophoblast invasion. Blastocyst-like spheroids formed by BeWo cells exhibited invasiveness and migration capabilities in differentiated EnSCs in our ‘*in vitro* implantation’ model, but this was severely restricted in CDC42-deficient EnSCs with decidualization induction (Fig. 4K and L). Hence, we have clearly demonstrated that CDC42 deficiency in EnSCs negatively affects the decidualization response and the invasion of trophoblast cells, which may lead to implantation failure.

### CDC42 enzymatic activity inhibition leads to decidualization impairment without inducing cellular senescence

As a Rho-GTPase, the enzymatic activity of CDC42 plays an important role in its physiological functions. To investigate whether the role of CDC42 in regulating stromal cell senescence and decidualization depends on its enzymatic activity, we used the CDC42 enzymatic activity inhibitor ML141 in our studies (Chaker *et al.*, 2018). We noticed that, under bright-field microscopy, ML141-treated primary EnSCs remained spindle-shaped fibroblast-like cells after decidualization stimulation, without an obvious change in cell size (Supplementary Fig. S3A–C), which was further verified by F-actin immunofluorescence staining (Supplementary Fig. S3D). In addition, after ML141 treatment, the secretion of PRL and IGFBP1 was significantly decreased (Supplementary Fig. S3E and F), indicating impaired stromal cell decidualization after CDC42 enzymatic activity inhibition. However, ML141 treatment neither exacerbated SA-β-gal staining during decidualization (Supplementary Fig. S3G and H), nor induced the upregulation of CDC42 and aging-related molecules CDKN2A, CDKN1A, and IL6 (Supplementary Fig. S3I–L), but significantly downregulated the expression of CXCL8 (Supplementary Fig. S3M). In addition, ML141 treatment hindered the upregulation of sST2 without affecting the secretion of CLU (Supplementary Fig. S3N and O). Thus, simply inhibiting CDC42 activity without changing the expression of CDC42 could hinder the decidualization progression of EnSCs, but could not lead to cellular senescence. To summarize, CDC42 enzymatic activity was required for the decidual response of EnSCs, but not for CDC42-regulated EnSC senescence.

### Transcriptomic analysis reveals WNT signaling activation in CDC42-knockdown stromal cells

**Figure 5.**
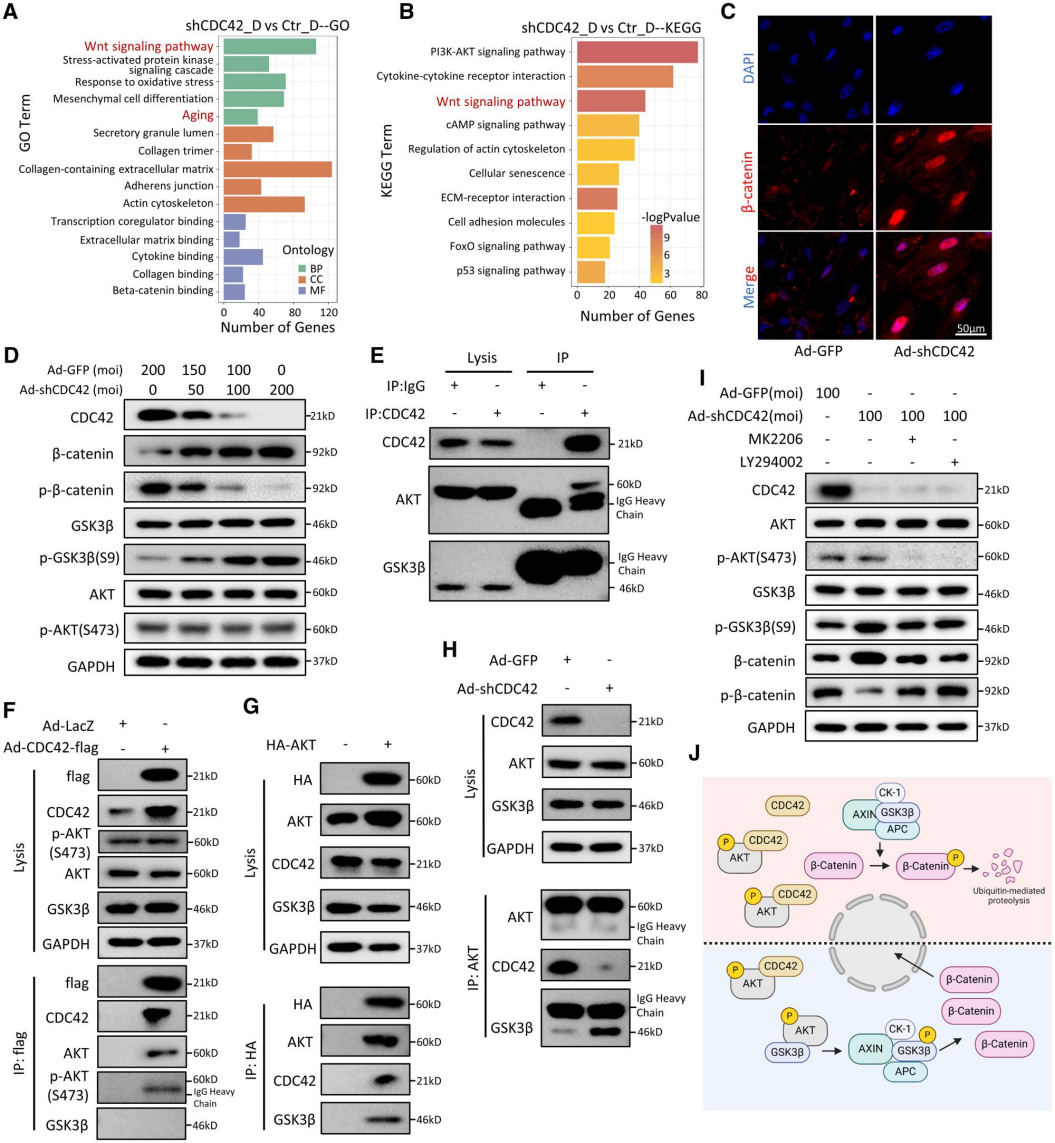
**CDC42 modulates Wnt signaling by interfering with the GSK3β-AKT interaction.** (**A**) Gene ontology (GO) enrichment of the differential expressed genes (DEGs) between Ctr_D (control) endometrial stromal cells (EnSCs) and shCDC42_D (CDC42 knockdown) EnSCs. BP, biological process; CC, cellular component; MF, molecular function. (**B**) Kyoto Encyclopedia of Genes and Genomes (KEGG) enrichment of the DEGs between Ctr_D EnSCs and shCDC42_D EnSCs. (**C**) Representative images of β-catenin immunofluorescence staining in primary EnSCs after 72 h of adenovirus treatment. (**D**) Expressions of β-catenin, p-β-catenin (Ser33/37/Thr41), GSK3β, p-GSK3β(S9), AKT, and p-AKT(S473) protein levels in primary EnSCs after 72 h of adenovirus treatment. (**E**) Extracts from T-EnSCs were subjected to immunoprecipitation with CDC42 antibody or Rabbit IgG control, then for western-blot analysis of CDC42, GSK3β, and AKT. (**F**) Extracts from T-EnSCs treated with Ad-CDC42-flag or Ad-LacZ adenovirus were subjected to immunoprecipitation with flag-beads, then for western-blot analysis of flag, CDC42, GSK3β, AKT, and p-AKT(S473). (**G**) Extracts from T-EnSCs transfected with HA-AKT plasmid or vector plasmid were subjected to immunoprecipitation with HA-beads, then for western-blot analysis of HA, AKT, CDC42, and GSK3β. (**H**) Extracts from T-EnSCs treated with Ad-GFP or Ad-shCDC42 adenovirus were subjected to immunoprecipitation with AKT antibody, then for western-blot analysis of AKT CDC42 and GSK3β. (**I**) Expression of AKT, p-AKT(S473), GSK3β, p-GSK3β(S9), β-catenin, and p-β-catenin (Ser33/37/Thr41) protein levels in T-EnSCs transfected with adenovirus following with or without 2 μM MK2206 or 25 μM LY294002. (**J**) Graphical illustration of the mechanism by which CDC42 regulates the activation of Wnt signaling in EnSCs. Adequate CDC42 protein in EnSCs interacts with p-AKT to protect p-AKT from binding and phosphorylating GSK3β, so the AXIN/APC/CK-1/GSK3β destruction complex induces the phosphorylation β-catenin following with ubiquitin-mediated proteolysis; with CDC42-knockdown in EnSCs, p-AKT is allowed to bind and phosphorylate GSK3β to inhibit GSK3β kinase activity, leading to the cytoplasmic accumulation and nuclear translocation of β-catenin for downstream transcription activation.

In order to investigate the mechanisms underlying EnSC senescence caused by CDC42 deficiency, we performed transcriptomic sequencing on three groups of primary EnSCs: control adenovirus group (Ctr), control adenovirus plus decidualization group (Ctr_D), and Ad-shCDC42 plus decidualization group (shCDC42_D). PCA and differential analysis revealed significant transcriptomic differences among these three groups of stromal cells (Supplementary Fig. S4A and B). GO enrichment of the DEGs suggested changes in ‘Aging’ in Biological Process (BP), as well as ‘Collagen-containing extracellular matrix’ and ‘Actin cytoskeleton’ in Cellular Component (CC) (Fig. 5A). KEGG analysis revealed pathways of ‘Regulation of actin cytoskeleton’ and ‘Cellular senescence’ enriched in the shCDC42_D group (Fig. 5B). GSEA and Heatmap analysis showed enrichment of genes associated with ‘Extracellular matrix organization’ in shCDC42_D compared with the Ctr_D group (Supplementary Fig. S4D and E). Increased reactive oxygen species (ROS) production has been regarded as a biomarker of aging (Aging Biomarker Consortium *et al.*, 2023). GO and GSEA suggested that ‘Response to oxidative stress’ was significantly enriched in the shCDC42_D group, which was confirmed by MitoSox staining showing the accumulation of intracellular ROS in shCDC42_D cells (Supplementary Fig. S4F and G). Since GO, KEGG, and GSEA analyses all included ‘Wnt signaling pathway’ (Fig. 5A and B, Supplementary Fig. S4C), we speculated that Wnt signaling is involved in CDC42-regulated EnSC senescence. Immunofluorescence staining of β-catenin, the key functional effector molecule of Wnt signaling, revealed that CDC42 knockdown induced β-catenin accumulation and translocation into the nucleus for downstream transcriptional regulation (Fig. 5C). GSK3β, a master regulator in Wnt/β-catenin signaling, phosphorylates cytoplasmic β-catenin for subsequent ubiquitin-mediated degradation. Meanwhile, phosphorylation at Serine 9 of GSK3β is the most common mechanism of GSK3β inactivation (Matsuda *et al.*, 2008). We found that CDC42-knockdown decreased the phosphorylation and degradation of β-catenin, probably by upregulating the phosphorylation of GSK3β (Fig. 5D). Collectively, our transcriptomic data showed that CDC42 knockdown facilitated EnSC senescence, which may be attributed to activated Wnt signaling.

### CDC42 modulates GSK3β phosphorylation by competitively interacting with AKT

Phosphorylation at Serine 9 of GSK3β is mainly mediated by AKT (Wakatsuki *et al.*, 2011), while PI3K-AKT signaling was enriched in our KEGG analysis. Western blot showed that knockdown of CDC42 had no effect on the level of both AKT and p-AKT-S473 (Fig. 5D), suggesting that CDC42 did not modulate Wnt signaling by activating AKT directly. Since a previous study reported the co-localization of CDC42 and phosphorylated AKT in mammalian fibroblasts (Higuchi *et al.*, 2001), we assumed an interaction among CDC42, AKT, and GSK3β in EnSCs. A co-immunoprecipitation (Co-IP) assay showed that endogenous CDC42 interacted with endogenous AKT in EnSCs, while no interaction was observed between CDC42 and GSK3β (Fig. 5E). Exogenously overexpressed CDC42 via Ad-CDC42-flag adenovirus had no impact on the expression of GSK3β, AKT, and p-AKT-S473. Co-IP with flag-beads showed the interaction between AKT, p-AKT-S473, and flag-labeled CDC42 in EnSCs (Fig. 5F). On the other hand, exogenously overexpressed AKT via HA-AKT plasmid and Co-IP with HA-beads revealed that AKT interacted with both CDC42 and GSK3β in EnSCs (Fig. 5G). Furthermore, CDC42 knockdown impeded the interaction between CDC42 and AKT, but promoted interaction between GSK3β and AKT in EnSCs (Fig. 5H). We utilized an allosteric AKT inhibitor, MK2206 (Lopez-Chavez *et al.*, 2015; Paulus *et al.*, 2017), as well as a PI3K/AKT inhibitor, LY294002 (Hu *et al.*, 2020), to further confirm whether CDC42 regulated Wnt signaling activation in an AKT-dependent manner. Both MK2206 and LY294002 suppressed the increase in p-GSK3β-S9 caused by CDC42 knockdown by inhibiting the phosphorylation of AKT, and hence restored the phosphorylation of β-catenin (Fig. 5I). All the above data suggested that CDC42 modulates Wnt signaling by interfering with the GSK3β-AKT interaction in EnSCs (Fig. 5J).

### Blocking WNT signaling partially rescues senescence in CDC42-deficient EnSCs

**Figure 6.**
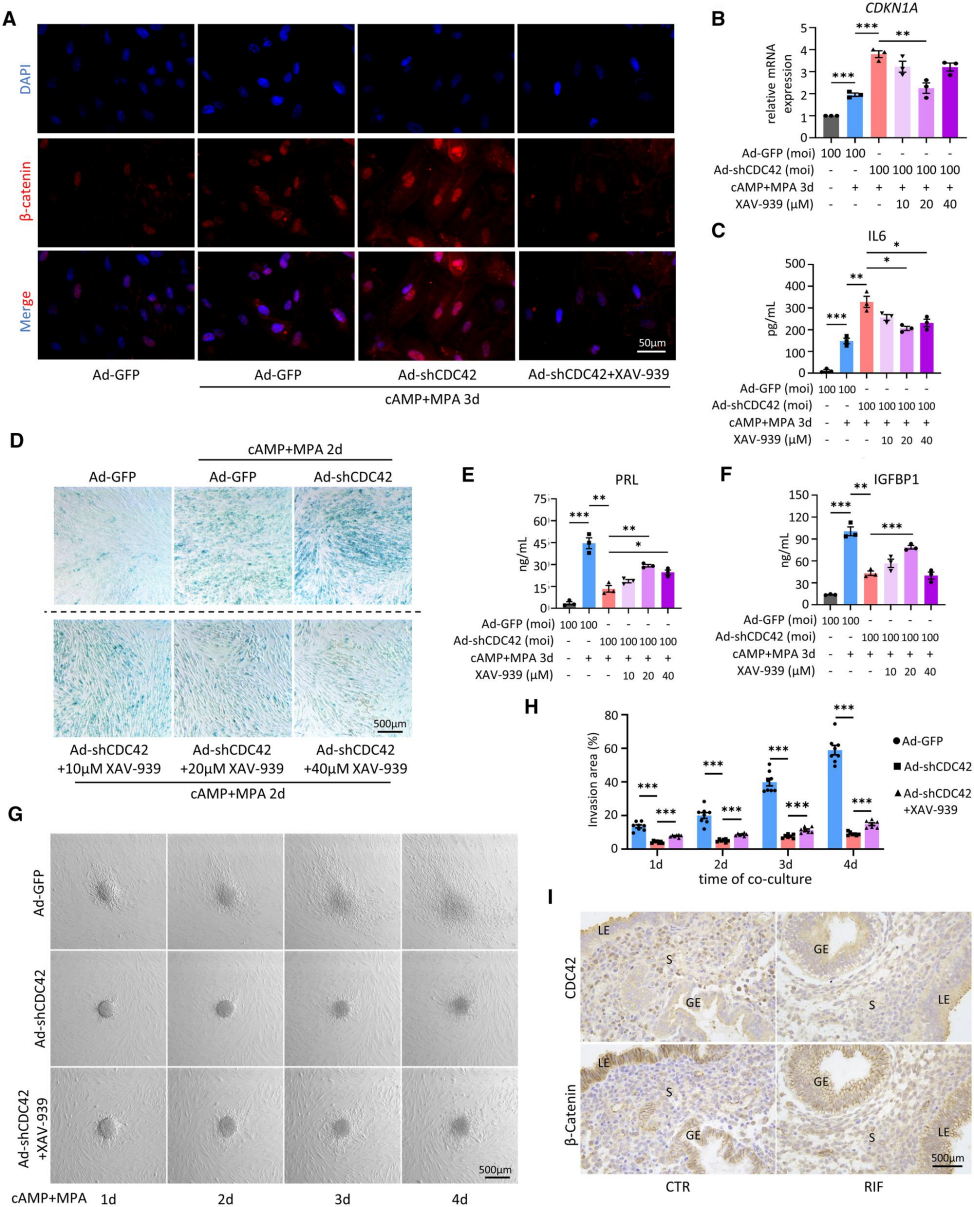
**Blocking Wnt signaling partially rescues senescence in CDC42-deficient endometrial stromal cells (EnSCs).** (**A**) Representative images of β-catenin immunofluorescence staining in immortalized EnSCs (T-EnSCs) transfected with Ad-GFP or Ad-shCDC42-GFP following with 72 h treatment of 8Br-cAMP+MPA with or without 20 μM XAV-939. (**B**) Expression of *CDKN1A* mRNA levels in T-EnSCs transfected with Ad-GFP or Ad-shCDC42-GFP following with 72 h treatment of 8Br-cAMP+MPA with or without 10, 20, or 40 μM XAV-939. (**C**) Level of IL6 secretion in T-EnSCs transfected with Ad-GFP or Ad-shCDC42-GFP following with 72 h treatment of 8Br-cAMP+MPA with or without 10, 20, or 40 μM XAV-939. (**D**) SA-β-gal staining of T-EnSCs transfected with Ad-GFP or Ad-shCDC42-GFP following with 48 h treatment of 8Br-cAMP+MPA with or without 10, 20, or 40 μM XAV-939. (**E** and **F**) Level of PRL and IGFBP1 secretion in T-EnSCs transfected with Ad-GFP or Ad-shCDC42-GFP following with 72 h treatment of 8Br-cAMP+MPA with or without 10, 20, or 40 μM XAV-939. (**G** and **H**) Representative bright filed images of different days after Bewo spheroids invaded the T-EnSCs transfected with Ad-GFP or Ad-shCDC42-GFP following with 48 h treatment of 8Br-cAMP+MPA with or without 20 μM XAV-939. Quantitative analysis of invasion area of Bewo spheroids. (**I**) Adjacent images of immunohistochemical staining of CDC42 and β-catenin in mid-secretory endometrium of control (CTR) and recurrent implantation failure patients. LE, luminal epithelium; GE, glandular epithelium; S, stroma. Mean±SEM. *P<0.05, **P<0.01, ***P<0.001. ANOVA with Tukey’s multiple comparisons test.

To further clarify the role of Wnt signaling in EnSC senescence and decidualization, we utilized a Wnt signaling inhibitor XAV-939, which stabilizes Axin-GSK3β complex by inhibiting TNKS activity and promotes the degradation of β-catenin (Huang *et al.*, 2009; Wang *et al.*, 2024). Immunofluorescence analysis revealed that XAV-939 treatment significantly inhibited the cytoplasmic accumulation and nuclear translocation of β-catenin caused by CDC42 knockdown in EnSCs (Fig. 6A). Different concentrations of XAV-939 treatment showed that 20 μM XAV-939 most effectively suppressed the elevation of *CDKN1A*, IL6, and CLU (Fig. 6B and C, Supplementary Fig. S5A and B). SA-β-gal staining showed that XAV-939 ameliorated senescence caused by CDC42 deficiency in a dose-dependent manner, but 40 μM XAV-939 had adverse effects on cell viability (Fig. 6D). Additionally, XAV-939 partially restored the mRNA expression and protein secretion of PRL and IGFBP1 (Fig. 6E and F, Supplementary Fig. S5B and C), and 20 μM XAV-939 exhibited the most significant amelioration. In the ‘*in vitro* implantation’ model, 20 μM XAV939 also significantly enlarged the invasion area of BeWo spheroids (Fig. 6G and H), suggesting that inhibition of Wnt signaling pathway could partially improve the decidual function impairment of EnSCs caused by CDC42 downregulation. Moreover, IHC assay of adjacent slices showed that, compared with CTR patients, the downregulation of CDC42 in mid-secretory RIF endometrial stroma was accompanied by nuclear translocation of β-catenin, further validating the role of Wnt activation in RIF endometrial senescence (Fig. 6I). Taken together, our findings indicate that Wnt signaling inhibitors showed potential for rescuing the stromal senescence, decidualization impairment, and embryo implantation failure caused by CDC42 deficiency, providing new insights in terms of the clinical interventions to improve the clinical pregnancy rate of RIF patients.

## Discussion

In this study, we observed a remarkable cellular senescence along with CDC42 deficiency in the endometrial stroma of RIF patients. CDC42 knockdown induced premature senescence of EnSCs, leading to aberrant accumulation of senescent EnSC during decidualization, which impaired the decidualization response and acceptance of trophoblast invasion. Transcriptomic analysis revealed activation of Wnt signaling in CDC42-deficient EnSCs. Further mechanistic studies suggested that CDC42 interacted with AKT competitively to impede the binding of GSK3β to AKT, leading to the reduction of phosphorylated GSK3β and subsequent β-catenin degradation. Lastly, we demonstrated that modulating cellular senescence by inhibiting aberrant Wnt signaling in CDC42-deficient EnSCs was a promising target for decidualization defects and embryo implantation failure.

Decidualization is a crucial step in the establishment of endometrial receptivity. Previous single-cell transcriptomic sequencing studies have demonstrated that during normal decidualization, a small population of senescent EnSCs promotes early inflammatory reactions and initiates decidualization (Lucas *et al.*, 2020). These senescent EnSCs are subsequently eliminated by uNK cells to establish an anti-inflammatory microenvironment, ensuring proper decidualization development (Brighton *et al.*, 2017). A disruption in endometrial immunity may impact the regulation of endometrial senescence. Immune-related infertility conditions such as antiphospholipid syndrome have been found to cause endometrial senescence (Tong *et al.*, 2022). In the later stages of decidualization, if embryo implantation does not occur, a significant number of stromal cells undergo senescence, contributing to endometrial shedding and menstruation (Muter *et al.*, 2023). However, there is limited research on whether excessive senescence contributes to infertility-related diseases, with existing studies primarily using transcriptomic sequencing analyses (Lucas *et al.*, 2020; Chen *et al.*, 2022; Zhao *et al.*, 2022).

Our study provides novel clinical evidence for a substantial correlation between stromal senescence in the endometrium of RIF, expanding our understanding of the physiological role of decidual senescence into pathological conditions. RIF is widely recognized as a heterogenous disease with various etiologies (Franasiak *et al.*, 2021). Although we have elucidated the significant role played by CDC42 in regulating endometrial senescence and receptivity, we have to admit that the CDC42 deficiency only accounts for a subset of RIF patients, which is clearly shown in Fig. 3I. According to a large transcriptomic sequencing study of Chen *et al.* (2022), 61.7% of young RIF patients (younger than 35 years old) showed endometrial aging, while the rest 38.3% suffered from other endometrial issues (e.g. immune active). In addition, as shown in Fig. 3K, although women with RIF had lower CDC42 expression, there was no significant negative correlation between CDC42 expression and number of embryos transferred, suggesting that endometrial receptivity is not determined by one single molecule. Therefore, the complex etiology of RIF needs to be comprehensively assessed by the combination of multiple molecules.

Notably, we also observed relatively mild senescence in the epithelial cells of both CTR and RIF endometria, aligning with previous reports that P16-positive epithelial cells in secretory glands account for only 2–3% of endometrial cells during the implantation window (Brighton *et al.*, 2017; Muter *et al.*, 2023). We believe that epithelial senescence is also under precise regulation, while disordered senescence of glandular epithelium may disturb the secretion of the glands to affect the microenvironment and early development of the embryo, imposing a higher risk of biochemical pregnancy and recurrent pregnancy loss. On the other hand, increased stromal senescence leads to endometrial fibrosis, which may be a major contributor to infertility of RIF patients. Interestingly, a recent study has reported that pharmacological elimination of acute senescence via dasatinib treatment at the time of decidualization stimulation inhibited embryo invasion (Rawlings *et al.*, 2021), which seemed contradictory to our results. In physiological conditions, acute senescence happens during the onset of decidualization stimulation, facilitates programmed tissue remodeling and embryo invasion, but would be promptly cleared by immune cells (Brighton *et al.*, 2017). However, our study revealed an obvious amplification of senescent EnSCs before decidualization stimulation caused by CDC42 knockdown, which persisted and further increased after decidualization stimulation. Similarly, Deryabin and Borodkina (2022) generated oxidative stress-induced senescent EnSCs and found that premature senescence of EnSCs impaired endometrial decidualization and hindered the interaction with trophoblast cells. We assume that senescent EnSCs probably accumulate during the proliferative phase (Tomari *et al.*, 2020; Deryabin and Borodkina, 2022), causing stromal fibrosis and implantation failure in mid-secretory endometrium of RIF patients. Besides, aberrant epithelial senescence and acute senescence of EnSCs may disturb embryo biosensing and selection, and promote the invasion of low-quality embryos, leading to (recurrent) pregnancy loss (Muter *et al.*, 2023). Further investigations are needed to clarify the possible roles of epithelial or stromal senescence in regulating female fertility.

The general characteristics of senescent cells include cell cycle arrest, increased cell size, metabolic disorder, telomere shortening, and accumulation of intracellular damage (Ogrodnik, 2021). Additionally, senescent cells exhibit an altered secretion spectrum and epigenetic changes (van Deursen, 2014; Gorgoulis *et al.*, 2019). Currently, it is widely accepted among researchers that no single marker is uniquely or specifically indicative of cellular senescence, and multiple markers should be employed to identify senescent cells (Gorgoulis *et al.*, 2019). The work by Lucas *et al.* (2020) shows that in the physiological situation, obvious differentiation of senescent stromal cells does not occur before 4 days of cAMP+MPA treatment. In this study, we employed a 3-day decidualization protocol, which induced little senescence in control group but significant senescence in CDC42 knockdown group, validating that CDC42 deficiency can cause premature senescence of endometrial stroma. The senescence phenotype of EnSCs following CDC42 knockdown was defined by the larger cell size, cell cycle arrest (P16, P21, and P53), DNA damage (p-γ-H2AX), and elevated SA-β-gal activity levels, as well as expression of SASP molecules (IL-6, IL-1α, IL-1β, and TGFB1), elucidating the crucial role played by CDC42 in regulating endometrial stromal senescence. It is worth noting that both IL-6 and IL-8 (CXCL8) are considered classical SASP molecules. Our study revealed that gradient knockdown of CDC42 during non-decidualization conditions resulted in a corresponding gradient increase in mRNA levels of IL-6 and CXCL8. During the decidualization condition, the level of IL-6 increased in CDC42-deficient EnSCs, while the level of CXCL8 was decreased. Similar findings have been reported in studies investigating decidual senescence induced by anti-cardiolipin antibodies, in which inflammatory responses might exert a greater influence on IL-8 during decidualization compared with cellular senescence (Tong *et al.*, 2022). Therefore, further investigations are required to determine whether the expression level of IL-8 (CXCL8) during decidualization can serve as an indicator for endometrial senescence.

Currently, numerous studies have reported the occurrence of lung fibrosis (Yao *et al.*, 2021), liver fibrosis (Udomsinprasert *et al.*, 2021), and kidney fibrosis due to cell senescence (Li *et al.*, 2021). The substantial secretion of SASP by senescent cells is a significant contributor to fibrosis (Maus *et al.*, 2023), resulting in tissue and organ dysfunction. In this study, we demonstrated that knockdown of CDC42 induced EnSC senescence and increased expression of various collagen *in vitro*. Meanwhile, CDC42 expression in endometrial stroma was negatively associated with stromal senescence and fibrosis in RIF patients. We speculate that both the decidualization defect and fibrosis caused by CDC42 deficiency contribute to restricted BeWo spheroids invasion *in vitro* and embryo implantation failure *in vivo*. Previous research has also highlighted the association between downregulation of CDC42 and tissue/organ fibrosis. Reduced levels of CDC42 in alveolar stem cells contributed to progressive pulmonary fibrosis (Wu *et al.*, 2020). Mice with specific knockdown of Cdc42 in renal tubular epithelial cells exhibited increased fibrotic changes in distal tubules and collecting ducts (Choi *et al.*, 2013). Downregulation of CDC42 in mouse fallopian tube epithelial cells led to type I collagen deposition within the fallopian tube (Jiang *et al.*, 2022). Our findings further confirm that CDC42 plays a pivotal role in regulating cellular senescence and tissue fibrosis within the endometrial stroma, thereby emphasizing its significance within the field.

However, some studies have revealed a different role of CDC42 in cellular senescence. Pharmacological inhibition of CDC42 activity functionally rejuvenates aged hematopoietic stem cells (Florian *et al.*, 2012). Elevated Cdc42-GTP level caused by gene targeting CDC42 GTPase-activating protein promotes a premature cellular senescence phenotype in mice, while pharmacological inhibition of CDC42 activity extends lifespan in aged female mice (Wang *et al.*, 2007; Florian *et al.*, 2020). Our previous study, as well as a report from other researchers, has proven that CDC42 can play a role independent of its Rho-GTPase activity, in which CDC42 binds p110β isoform via its RAS-binding domain to activate PI3K-AKT signaling (Fritsch *et al.*, 2013; Jiang *et al.*, 2022). In this study, we have demonstrated that CDC42 regulates endometrial senescence through modulating the interaction among CDC42/AKT/GSK3β complex in a protein level-dependent way. On the other hand, inhibition of CDC42 activity via ML141 impaired cytoskeleton remodeling in decidualization without changing the CDC42 expression level and cellular senescence level, which is totally understandable since CDC42 controls the organization of the actin cytoskeleton dependent on its GTPase activity (Tapon and Hall, 1997). Thus, during endometrial decidualization, CDC42 plays two vital roles: regulating cytoskeleton remodeling in a Rho-GTPase activity-dependent way, and regulating cellular senescence in an expression level-dependent way via interacting with AKT protein. A recently published spatial and single-cell atlas of the mouse female reproductive tract revealed that cyclic remodeling may give the uterus different properties in cell and organ senescence (Winkler *et al.*, 2024). The unique periodical change of the endometrium, especially the cyclic proliferation, decidual differentiation, and programmed shedding of the stroma, probably accounts for the special regulatory mechanism of CDC42 in this tissue. Therefore, the detailed role of CDC42 expression and CDC42 activity in endometrial senescence needs to be further explored in mice and humans in the future.

The Wnt/β-catenin signaling pathway is highly conserved throughout evolution and plays a crucial role in regulating various fundamental biological processes (Logan and Nusse, 2004; Clevers and Nusse, 2012). In recent years, numerous studies have reported the association between Wnt signaling activation and senescence. For instance, persistent Wnt activation has been observed in a Klotho mouse model with accelerated aging, leading to stem cell depletion and senescence-related changes (Liu *et al.*, 2007). Increased Wnt signaling has been detected in the lungs of elderly patients, resulting in A-TII cell senescence and impaired progenitor cell function (Lehmann *et al.*, 2020). Ectopic expression of Wnt1 induces mitochondrial dysfunction and cellular senescence in human proximal tubule cells (HKC-8) (Miao *et al.*, 2019). Furthermore, aberrant activation of the classical Wnt pathway has been implicated in various fibrotic diseases, serving as a common feature of systemic fibrotic disorders (e.g. systemic sclerosis) as well as isolated organ fibrosis (e.g. lung, kidney, or liver fibrosis) (Akhmetshina *et al.*, 2012). Herein, we demonstrate that overactivation of Wnt signaling mediates EnSC senescence and fibrosis as a result of downregulation of CDC42. The abnormal decidualization and compromised trophoblast invasion can be rescued by Wnt signaling inhibitor XAV-939 in a dose-dependent manner. However, further comprehensive clinical trials are required to investigate whether inhibition of excessive Wnt activation can improve endometrial receptivity and pregnancy outcomes for RIF patients. In addition, it was noted that XAV-939 treatment only showed a partial rescue in CDC42-deficient EnSCs. Disturbance of other signaling pathways, such as AKT, FoxO, and p53 pathways (shown in Fig. 5B) may be involved in the process, and needs to be explored in the future.

There are some limitations of this study. Firstly, the present study was based on *in vitro* cell cultures and neglected the interactions between different cellular compartments. Further studies involving CDC42-regulated endometrial senescence are needed in conditional knockout mice model or human endometrial assembloids. Secondly, although shRNA has been reported to produce less off-target transcription regulation than corresponding siRNA (Rao *et al.*, 2009), we have to be careful about the possibility of off-target effects on cell senescence. Generating adenovirus harboring shRNA with mismatch mutation in positions 9–11 as the control adenovirus (Buehler *et al.*, 2012), combining shRNA with genetic mutation via CRISPR/cas9 (Peretz *et al.*, 2018), or co-delivering inhibitory tough decoy RNAs (Mockenhaupt *et al.*, 2015) may help solve this problem.

In addition to uncovering endometrial senescence in the pathological condition of RIF, our study provides the biological evidence for the indispensable function of CDC42 in regulating EnSC senescence and decidualization by modulating the Wnt/β-catenin signaling pathway. CDC42 competes with GSK3β for binding to AKT, thus ensuring β-catenin degradation by the Axin-GSK3β destruction complex. These findings highlight the importance of CDC42 in maintaining proper endometrial function and suggest its potential as a therapeutic target for improving endometrial receptivity and embryo implantation in RIF patients.

## Supplementary Material

deae246_Supplementary_Figure_S1

deae246_Supplementary_Figure_S2

deae246_Supplementary_Figure_S3

deae246_Supplementary_Figure_S4

deae246_Supplementary_Figure_S5

deae246_Supplementary_Table_S1

deae246_Supplementary_Table_S2

deae246_Supplementary_Table_S3

## Data Availability

Detailed description of methods and original data of this study are available from the authors upon reasonable request. RNA-seq data sets generated in this study have been deposited at the NCBI database with BioProject accession number PRJNA1102745.
