# Supplementary material for: CDC42 deficiency leads to endometrial stromal cell senescence in recurrent implantation failure
Source: Hum Reprod. 2024 Nov 1;39(12):2768–84. doi: 10.1093/humrep/deae246 (PMC11630066; doi:10.1093/humrep/deae246)
Supplement: deae246_Supplementary_Figure_S1 [file deae246_supplementary_figure_s1.pdf]

Human Reproduction, 2024, 1–1
https://doi.org/10.1093/humrep/deae246
Supplementary Data

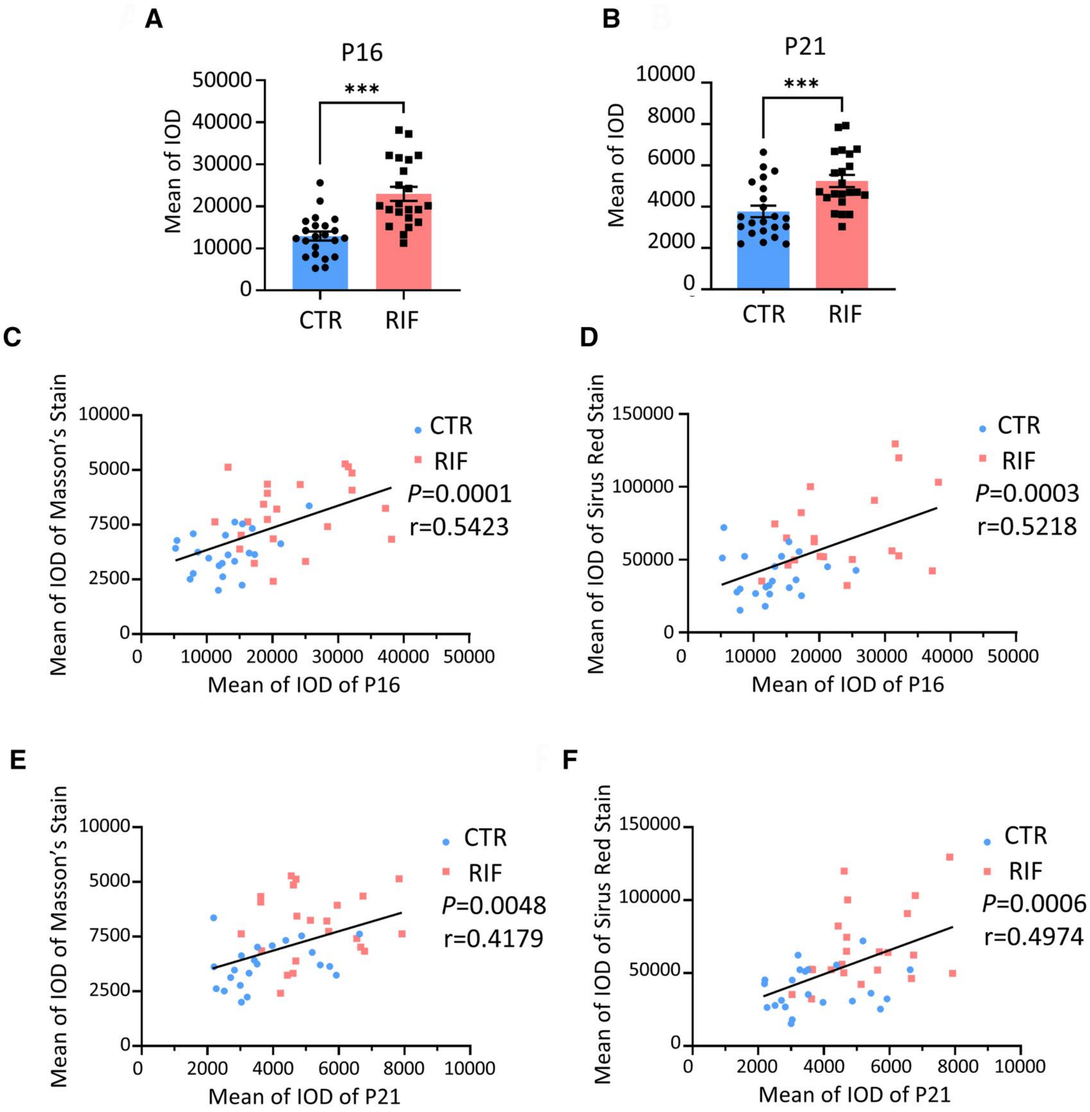

Supplementary Figure S1. Endometrial fibrosis is associated with senescence. (**A**) Quantitative analysis of integrated optical density (IOD) for immunohistochemical (IHC) staining of P16 in mid-secretory endometrium of CTR patients (n = 22) versus recurrent implantation failure (RIF) patients (n = 22). (**B**) Quantitative analysis of IOD for IHC staining of P21 in mid-secretory endometrium of CTR patients (n = 22) versus RIF patients (n = 22). (**C**) Linear regression analysis of the correlation between P16 and Masson's staining in Supplementary Fig. S1A and Fig. 1L. (**D**) Linear regression analysis of the correlation between P16 and Sirus Red staining in Supplementary Fig. S1A and Fig. 1N. (**E**) Linear regression analysis of the correlation between P21 and Masson's staining in Supplementary Fig. S1B and Fig. 1L. (**F**) Linear regression analysis of the correlation between P21 and Sirus Red staining in Supplementary Fig. S1B and Fig. 1N. Mean±SEM. ***P<0.001. Student's t-test.
