# Supplementary material for: CDC42 deficiency leads to endometrial stromal cell senescence in recurrent implantation failure
Source: Hum Reprod. 2024 Nov 1;39(12):2768–84. doi: 10.1093/humrep/deae246 (PMC11630066; doi:10.1093/humrep/deae246)
Supplement: deae246_Supplementary_Figure_S2 [file deae246_supplementary_figure_s2.pdf]

OXFORD
human reproduction
Human Reproduction, 2024, 1–1
https://doi.org/10.1093/humrep/deae246
Supplementary Data

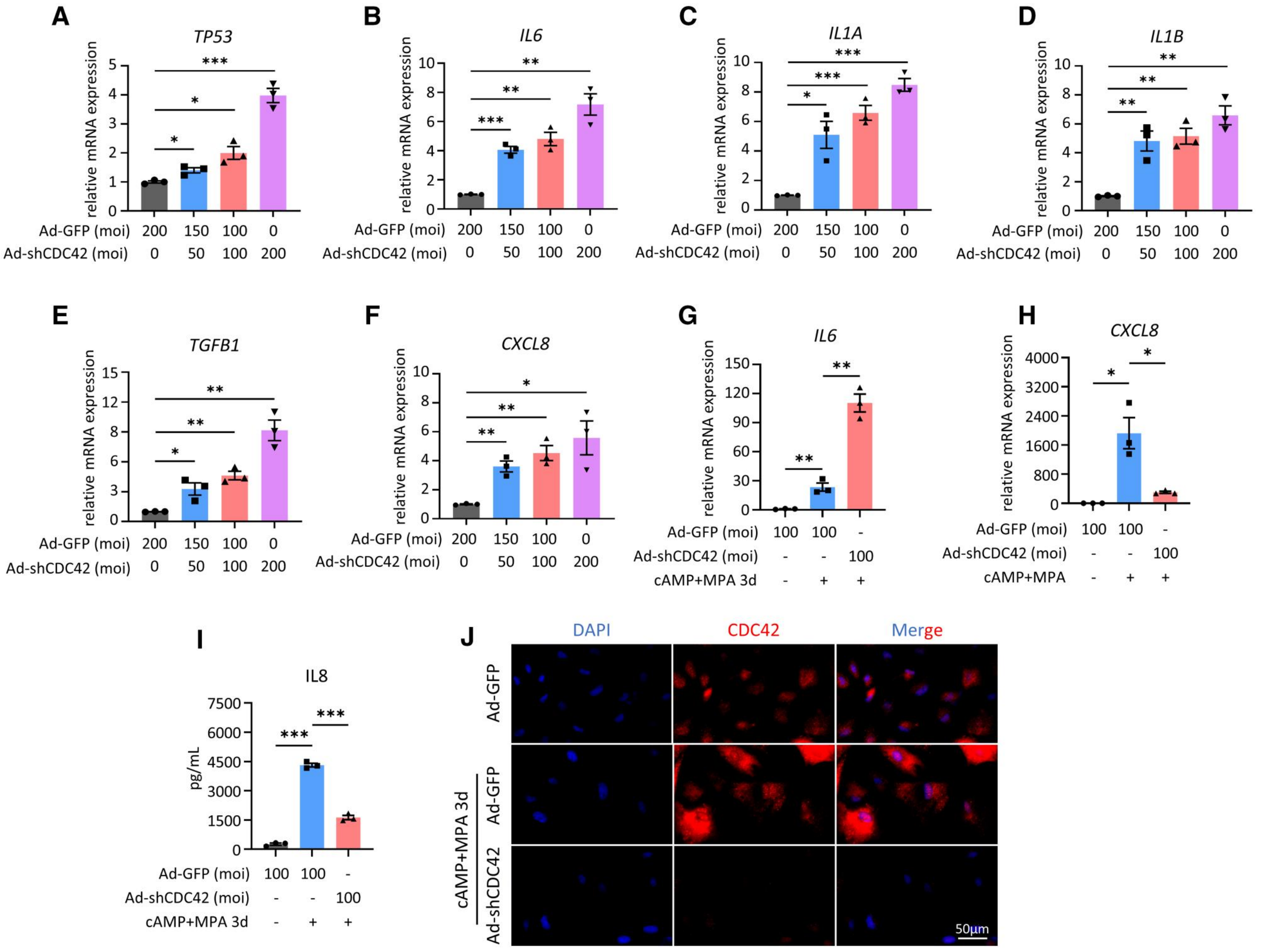

Supplementary Figure S2. CDC42 knockdown promotes senescence in endometrial stromal cells (EnSCs). (**A**–**F**) Expression of *TP53*, *IL6*, *IL1A*, *IL1B*, *TGFB1*, and *CXCL8* mRNA levels in primary EnSCs after 72 h of adenovirus treatment. (**G** and **H**) Expression of *IL6* and *CXCL8* mRNA levels in primary EnSCs transfected with Ad-GFP or Ad-shCDC42-GFP following with 72 h of 8Br-cAMP+MPA treatment. (**I**) Level of IL8 secretion in primary EnSCs transfected with Ad-GFP or Ad-shCDC42-GFP following with 72 h of 8Br-cAMP+MPA treatment. (**J**) Representative images of CDC42 immunofluorescence staining in primary EnSCs transfected with Ad-GFP or Ad-shCDC42-GFP following with 72 h of 8Br-cAMP+MPA treatment. Mean± SEM. *P<0.05, **P<0.01, ***P<0.001. ANOVA with Tukey's multiple comparisons test.
