# Supplementary material for: CDC42 deficiency leads to endometrial stromal cell senescence in recurrent implantation failure
Source: Hum Reprod. 2024 Nov 1;39(12):2768–84. doi: 10.1093/humrep/deae246 (PMC11630066; doi:10.1093/humrep/deae246)
Supplement: deae246_Supplementary_Figure_S3 [file deae246_supplementary_figure_s3.pdf]

OXFORD
human reproduction
Human Reproduction, 2024, 1–1
https://doi.org/10.1093/humrep/deae246
Supplementary Data

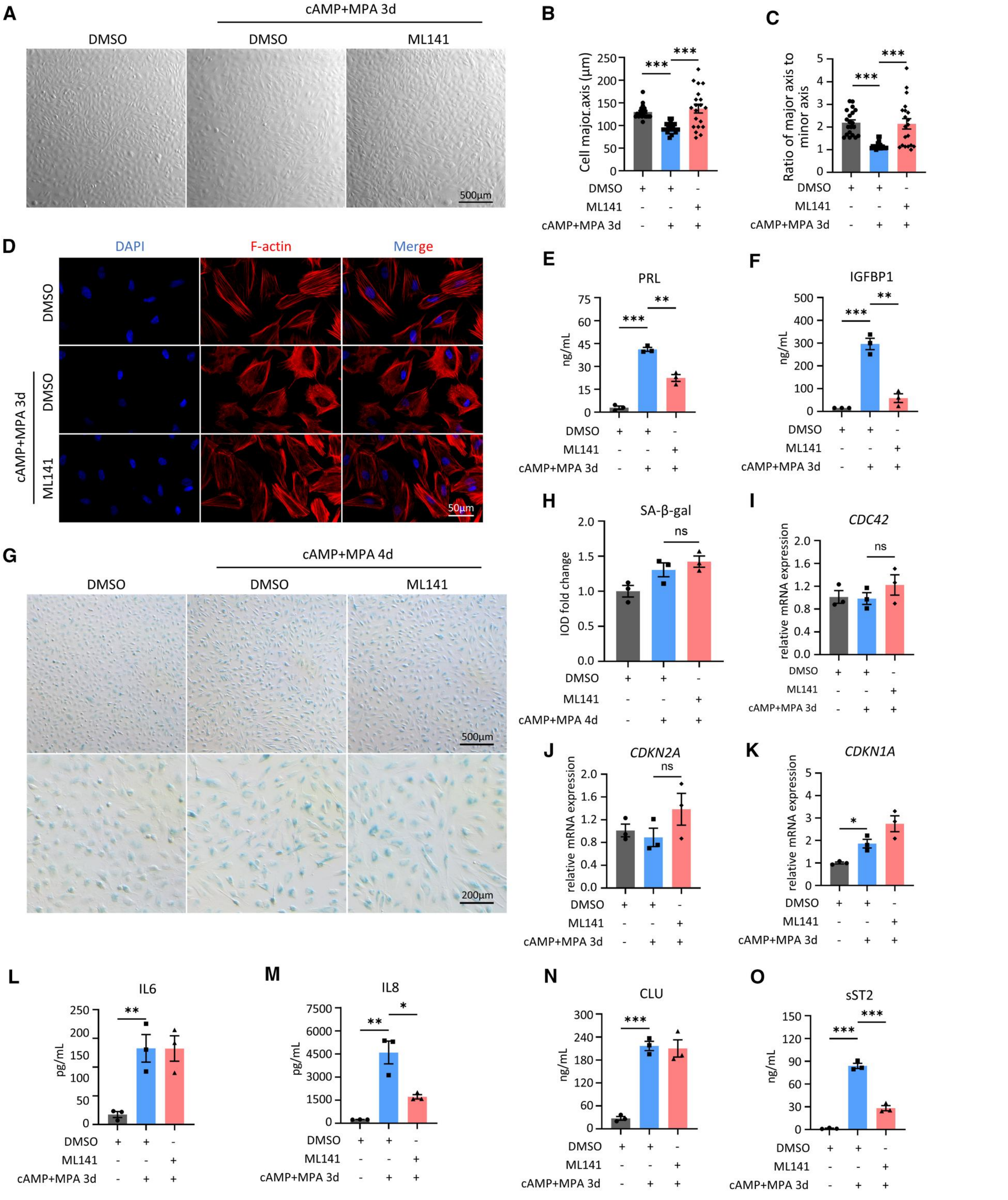

Supplementary Figure S3. CDC42 enzymatic activity inhibition led to decidualization impairment without inducing cellular senescence. (**A**–**C**) Representative bright field images of primary endometrial stromal cells (EnSCs) treated with DMSO or 10 μM ML141 following with 72 h of 8Br-cAMP+MPA treatment. Quantitative analysis of cell major axis and the ratio of cell major axis to minor axis. (**D**) Representative images of F-actin immunofluorescence staining in primary EnSCs treated with DMSO or 10 μM ML141 following with 72 h of 8Br-cAMP+MPA treatment, with continuous addition of inhibitors during decidualization. (**E** and **F**) Level of PRL and IGFBP1 secretion in primary EnSCs treated with DMSO or 10 μM ML141 following with 72 h of 8Br-cAMP+MPA treatment, with continuous addition of inhibitors during decidualization. (**G** and **H**) SA-β-gal staining of primary EnSCs treated with DMSO or 10 μM ML141 following with 96 h of 8Br-cAMP+MPA treatment, with continuous addition of inhibitors during decidualization. Fold change of integrated optical density for SA-β-gal staining. (**I**–**K**) Expression of *CDC42*, *CDKN2A*, and *CDKN1A* mRNA levels in primary EnSCs treated with DMSO or 10 μM ML141 following with 72 h of 8Br-cAMP+MPA treatment, with continuous addition of inhibitors during decidualization. Decidualization induction medium supplemented with DMSO or ML141 was replaced every day. (**L**–**O**) Level of IL6, IL8, CLU, and sST2 secretion in primary EnSCs treated with DMSO or 10 μM ML141 following with 72 h of 8Br-cAMP+MPA treatment, with continuous addition of inhibitors during decidualization. Mean±SEM. **P*<0.05, ***P*<0.01, ****P*<0.001. ns, not significant. ANOVA with Tukey's multiple comparisons test.
