# Supplementary material for: CDC42 deficiency leads to endometrial stromal cell senescence in recurrent implantation failure
Source: Hum Reprod. 2024 Nov 1;39(12):2768–84. doi: 10.1093/humrep/deae246 (PMC11630066; doi:10.1093/humrep/deae246)
Supplement: deae246_Supplementary_Figure_S4 [file deae246_supplementary_figure_s4.pdf]

Human Reproduction, 2024, 1–1
https://doi.org/10.1093/humrep/deae246
Supplementary Data

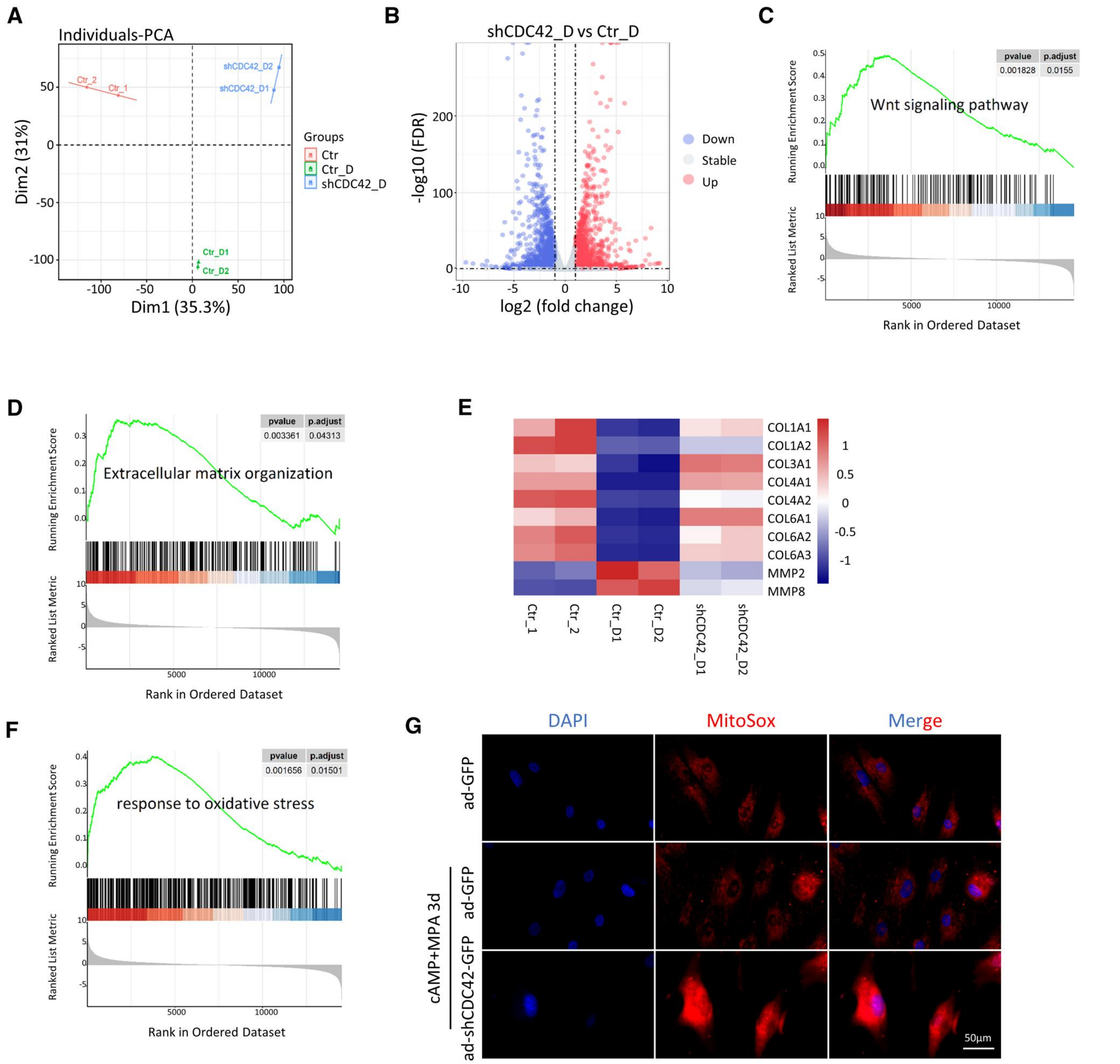

Supplementary Figure S4. Transcriptomic analysis of CDC42-knockdown stromal cells. (**A**) Principal component analysis of the gene expression profile of primary endometrial stromal cells (EnSCs) which received 48 h of ad-GFP treatment (Ctr group), 48 h of ad-GFP treatment and then 72 h of 8Br-cAMP+MPA treatment (Ctr_D group), 48 h of ad-shCDC42-GFP treatment and then 72 h of 8Br-cAMP+MPA treatment (shCDC42_D group). (**B**) Volcano plot of the differential expressed genes (DEGs) between Ctr_D EnSCs and shCDC42_D EnSCs. (**C**) Gene Set Enrichment Analysis (GSEA) of 'Wnt signaling pathway' enrichment in shCDC42_D EnSCs compared with Ctr_D EnSCs. (**D**) GSEA of 'Extracellular matrix organization' enrichment in shCDC42_D EnSCs compared with Ctr_D EnSCs. (**E**) Heatmap analysis showing the expression of genes associated with extracellular matrix organization. (**F**) GSEA of 'Response to oxidative stress' enrichment in shCDC42_D EnSCs compared with Ctr_D EnSCs. (**G**) Representative images of ROS levels detected by Mito-SOX staining in T-EnSCs transfected with Ad-GFP or Ad-shCDC42-GFP following with 72 h of 8Br-cAMP+MPA treatment.
