# Supplementary material for: CDC42 deficiency leads to endometrial stromal cell senescence in recurrent implantation failure
Source: Hum Reprod. 2024 Nov 1;39(12):2768–84. doi: 10.1093/humrep/deae246 (PMC11630066; doi:10.1093/humrep/deae246)
Supplement: deae246_Supplementary_Figure_S5 [file deae246_supplementary_figure_s5.pdf]

OXFORD
human reproduction
Human Reproduction, 2024, 1–1
https://doi.org/10.1093/humrep/deae246
Supplementary Data

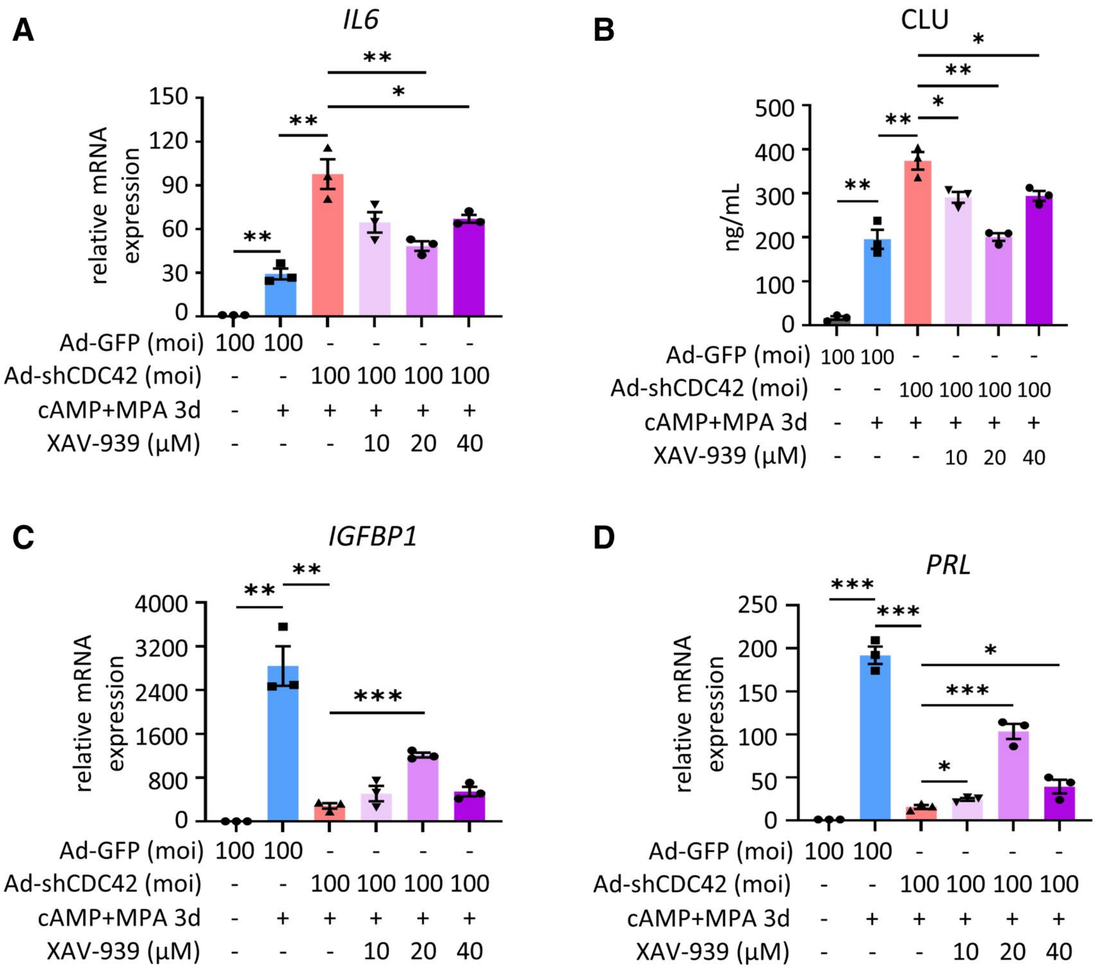

Supplementary Figure S5. **Blocking Wnt signaling partially rescues senescence and restores decidualization caused by CDC42 deficiency.** (**A**) Expression of *IL6* mRNA levels in immortalized endometrial stromal cells (T-EnSCs) transfected with Ad-GFP or Ad-shCDC42-GFP following with 72 h treatment of 8Br-cAMP+MPA with or without 10, 20, or 40 μM XAV-939. (**B**) Level of CLU secretion in T-EnSCs transfected with Ad-GFP or Ad-shCDC42-GFP following with 72 h treatment of 8Br-cAMP+MPA with or without 10, 20, or 40 μM XAV-939. (**C** and **D**) Expression of *PRL* and *IGFBP1* mRNA levels in T-EnSCs transfected with Ad-GFP or Ad-shCDC42-GFP following with 72 h treatment of 8Br-cAMP+MPA with or without 10, 20, or 40 μM XAV-939. Mean ±SEM. *P<0.05, **P<0.01, ***P<0.001. ANOVA with Tukey's multiple comparisons test.
