# Supplementary material for: CDC42 deficiency leads to endometrial stromal cell senescence in recurrent implantation failure
Source: Hum Reprod. 2024 Nov 1;39(12):2768–84. doi: 10.1093/humrep/deae246 (PMC11630066; doi:10.1093/humrep/deae246)
Supplement: deae246_Supplementary_Table_S1 [file deae246_supplementary_table_s1.pdf]

OXFORD

human reproduction

Human Reproduction, 2024, 1–1
https://doi.org/10.1093/humrep/deae246
Supplementary Data

Supplemental Table S1. Clinical characteristics of women recruited in the study.

Variables	CTR-proliferative (n = 12)	CTR-mid secretory (n = 59)	RIF-mid secretory (n = 37)	P-value
Age (years)	29.50 ± 0.66	30.02 ± 0.41	30.11 ± 0.43	0.801
BMI (kg/m^2)	21.57 ± 0.66	22.98 ± 0.50	22.15 ± 0.53	0.306
Basal FSH (mIU/ml)	7.08 ± 0.41	7.56 ± 0.28	7.17 ± 0.36	0.595
Basal LH (mIU/ml)	4.65 ± 0.56	5.54 ± 0.39	4.41 ± 0.44	0.136
Basal E_2 (pmol/ml)	44.99 ± 5.85	39.63 ± 2.15	44.26 ± 4.32	0.495
Infertility duration (years)	3.67 ± 0.67	3.00 ± 0.26	3.14 ± 0.27	0.534
Number of embryos transferred	1.83 ± 0.21	1.64 ± 0.09	5.78 ± 0.29	<0.001***

Values presented as mean±SEM. One-way ANOVA was employed to determine the difference among groups.

	For IHC assay			
Variables	CTR-proliferative (n = 6)	CTR-mid secretory (n = 28)	RIF-mid secretory (n = 22)	P-value
Age (years)	29.83 ± 0.98	30.61 ± 0.57	30.86 ± 0.52	0.719
BMI (kg/m^2)	22.37 ± 0.99	23.60 ± 0.59	22.85 ± 0.76	0.594
Basal FSH (mIU/ml)	6.75 ± 0.42	6.99 ± 0.31	7.52 ± 0.46	0.509
Basal LH (mIU/ml)	4.41 ± 0.49	5.05 ± 0.39	4.67 ± 0.69	0.799
Basal E_2 (pg/ml)	49.14 ± 10.22	36.95 ± 5.98	46.28 ± 6.85	0.487
Infertility duration (years)	3.17 ± 0.83	3.38 ± 0.35	3.21 ± 0.39	0.936
Number of embryos transferred	1.67 ± 0.33	1.79 ± 0.13	6.09 ± 0.41	<0.001***

Values presented as mean±SEM. One-way ANOVA was employed to determine the difference among groups.

	For WB/RT-qPCR assay			
Variables	CTR-proliferative (n = 6)	CTR-mid secretory (n = 18)	RIF-mid secretory (n = 12)	P-value
Age (years)	29.17 ± 0.95	29.11 ± 0.75	28.83 ± 0.59	0.954
BMI (kg/m^2)	20.77 ± 0.84	21.17 ± 0.62	20.82 ± 0.73	0.909
Basal FSH (mIU/ml)	7.42 ± 0.71	8.26 ± 0.57	6.98 ± 0.64	0.311
Basal LH (mIU/ml)	4.89 ± 1.05	5.74 ± 0.75	4.07 ± 0.44	0.248
Basal E_2 (pg/ml)	40.83 ± 6.24	47.46 ± 3.95	39.31 ± 2.72	0.293
Infertility duration (years)	4.17 ± 1.08	2.94 ± 0.50	3.33 ± 0.39	0.439
Number of embryos transferred	2.00 ± 0.26	1.50 ± 0.15	5.33 ± 0.48	<0.001***

Values presented as mean±SEM. One-way ANOVA was employed to determine the difference among groups.

	For primary EnSCs		
Variables	CTR-mid secretory (n = 13)	RIF-mid secretory (n = 3)	P-value
Age (years)	30.08 ± 0.79	29.67 ± 2.60	0.841
BMI (kg/m^2)	22.18 ± 0.94	22.30 ± 0.46	0.952
Basal FSH (mIU/ml)	7.42 ± 0.49	5.42 ± 0.83	0.093
Basal LH (mIU/ml)	5.66 ± 0.94	3.87 ± 1.39	0.408
Basal E_2 (pg/ml)	42.11 ± 4.87	49.99 ± 19.52	0.561
Infertility duration (years)	2.39 ± 0.35	1.83 ± 0.44	0.486
Number of embryos transferred	1.62 ± 0.21	5.33 ± 0.33	<0.001***

Values presented as mean±SEM. Student’s *t*-test was employed to determine the difference between groups.
RIF, recurrent implantation failure; IHC, immunohistochemical; EnSCs, endometrial stromal cells.
