# Supplementary material for: CDC42 deficiency leads to endometrial stromal cell senescence in recurrent implantation failure
Source: Hum Reprod. 2024 Nov 1;39(12):2768–84. doi: 10.1093/humrep/deae246 (PMC11630066; doi:10.1093/humrep/deae246)
Supplement: deae246_Supplementary_Table_S2 [file deae246_supplementary_table_s2.pdf]

Human Reproduction, 2024, 1–1
https://doi.org/10.1093/humrep/deae246
Supplementary Data

Supplemental Table S2. Details of antibodies used.

Antibody	Application (dilution)	Source	Catalogue number
CDC42	IHC (1:400), IF (1:200), WB (1:3000), IP (1:100)	Abcam	ab187643
P16	IHC (1:400), IF (1:200)	proteintech	10883-1-AP
P21	IHC (1:400), IF (1:200), WB (1:1000)	proteintech	10355-1-AP
P53	WB (1:1000)	proteintech	10442-1-AP
p-γH2AX	WB (1:500)	Abcam	ab22551
F-actin (Phalloidin-iFluor 594)	IF (1:1000)	Abcam	ab176757
MitoSOX Red	IF (0.25 μg/ml)	Invitrogen	M36008
FOXO1	WB (1:1000)	Cell Signaling Technology	97635
HOXA10	WB (1:500)	proteintech	26497-1-AP
Collagen I	WB (1:1000)	proteintech	14695-1-AP
Collagen III	WB (1:500)	proteintech	22734-1-AP
Collagen IV	WB (1:500)	Affinity	AF0510
MMP2	WB (1:500)	proteintech	10373-2-AP
β-catenin	IF (1:100), WB (1:1000), IHC (1:400)	Cell Signaling Technology	9562
p-β-catenin(Ser33/37/Thr41)	WB (1:1000)	Cell Signaling Technology	9561
GSK3β	WB (1:1000)	Cell Signaling Technology	9315
p-GSK3β(S9)	WB (1:1000)	Cell Signaling Technology	5558
AKT	WB (1:1000), IP(1:100)	Cell Signaling Technology	4691
p-AKT (S473)	WB (1:1000)	Cell Signaling Technology	9271
GAPDH	WB (1:10000)	Bioworld	AP0063
HRP-conjugated Goat anti Rabbit secondary antibody	WB (1:10000)	ZSGB-BIO	ZB-2301
HRP-conjugated Goat anti Mouse secondary antibody	WB (1:10000)	ZSGB-BIO	ZB-2305
Donkey anti-Rabbit Secondary Antibody, Alexa Fluor 594	IF (1:1000)	Invitrogen	A21207
Goat anti-Rabbit Secondary Antibody, Alexa Fluor 488	IF (1:1000)	Invitrogen	A11008
Rabbit IgG (H + L)	IP (1:500)	BOSTER	BA1045
anti-Flag-HRP	WB (1:10000)	Sigma	A8592
Anti-HA Tag Antibody	WB (1:1000)	Abm	G036

IHC, immunohistochemical.
