# Supplementary material for: CDC42 deficiency leads to endometrial stromal cell senescence in recurrent implantation failure
Source: Hum Reprod. 2024 Nov 1;39(12):2768–84. doi: 10.1093/humrep/deae246 (PMC11630066; doi:10.1093/humrep/deae246)
Supplement: deae246_Supplementary_Table_S3 [file deae246_supplementary_table_s3.pdf]

*Human Reproduction*, 2024, 1–1
https://doi.org/10.1093/humrep/deae246
Supplementary Data

**Supplementary Table S3.** Primers used for qRT-PCR (5′–3′).

| Gene | Forward primer | Reverse primer |
|---|---|---|
| 18S | CGGCTACCACATCCAAGGAA | CTGGAATTACCGCGGCT |
| CDC42 | CCCTCTACTATTGAGAAACTTG | AGAACACTCCACATACTTGA |
| CDKN2A | GGGTTTTCGTGGTTCACATCC | CTAGACGCTGGCTCCTCAGTA |
| CDKN1A | TGTCCGTCAGAACCCATGC | AAAGTCGAAGTTCCATCGCTC |
| TP53 | CAGCACATGACGGAGGTTGT | TCATCCAAATACTCCACACGC |
| IL6 | ACTCACCTCTTCAGAACGAATTG | CCATCTTTGGAAGGTTCAGGTTG |
| CXCL8 | TTTTGCCAAGGAGTGCTAAAGA | AACCCTCTGCACCCAGTTTTC |
| TGFB1 | TACCTGAACCCGTGTTGCTCTC | GTTGCTGAGGTATCGCCAGGAA |
| IL1A | TGTATGTGACTGCCCAAGATGAAG | AGAGGAGGTTGGTCTCACTACC |
| IL1B | CCACAGACCTTCCAGGAGAATG | GTGCAGTTCAGTGATCGTACAGG |
| PRL | GGAGCAAGCCCAACAGATGAA | GGCTCATTCCAGGATCGCAAT |
| IGFBP1 | TTTTACCTGCCAAACTGCAACA | CCCATTCCAAGGGTAGACGC |

© The Author(s) 2024. Published by Oxford University Press on behalf of European Society of Human Reproduction and Embryology.
This is an Open Access article distributed under the terms of the Creative Commons Attribution License (https://creativecommons.org/licenses/by/4.0/), which permits unrestricted reuse, distribution, and reproduction in any medium, provided the original work is properly cited.
Human Reproduction, 2024, 00, 1–25
https://doi.org/10.1093/humrep/deae246
Original Article
